# Bodies on Display: A Scoping Review of Appearance-Focused Social Media, Body Image, Self-Esteem, and Psychological Wellbeing in Adolescent Girls and Young Women

**DOI:** 10.3390/ejihpe16080108

**Published:** 2026-07-23

**Authors:** Giuseppe Marano, Senad Hasaj, Oksana Di Giacomi, Marco Lanzetta, Gabriele Sani, Marianna Mazza

**Affiliations:** 1 Department of Neuroscience, Head-Neck and Chest, Section of Psychiatry, Fondazione Policlinico, Universitario Agostino Gemelli IRCCS, Largo Agostino Gemelli 8, 00168 Rome, Italy; senadhasaj98@gmail.com (S.H.); oksanadigiacomi@gmail.com (O.D.G.);; 2 Department of Neuroscience, Section of Psychiatry, Università Cattolica del Sacro Cuore, Largo Agostino Gemelli, 00168 Rome, Italy

**Keywords:** social media, body image, self-esteem, psychological wellbeing, adolescents, young women, appearance-focused content, scoping review

## Abstract

Background: The widespread use of appearance-focused social media platforms has transformed how body-related attitudes are constructed, internalized, and evaluated, particularly among adolescent girls and young women, yet the existing literature remains heterogeneous in conceptual frameworks, populations, and outcome measures. Objective: This scoping review maps the extent, nature, and characteristics of the available evidence on the relationship between appearance-focused social media use, body image, self-esteem, and psychological wellbeing in adolescent girls and young women. Methods: Following PRISMA-ScR, PubMed, Scopus, and Web of Science were searched for quantitative, qualitative, and mixed-methods studies on female samples aged 10–30; data were charted and synthesized descriptively. Results: Fifty-eight studies (2014–2026) met the inclusion criteria. Evidence was concentrated in Australia and the United States and skewed toward emerging adults, with adolescent girls aged 10–17 examined in only nine studies. Appearance-focused engagement was consistently linked to body dissatisfaction, body surveillance, drive for thinness, and negative affect, with appearance comparison, internalization of appearance ideals, and self-objectification emerging as recurrent explanatory mechanisms. Body positive and body neutrality content emerged as the most promising protective categories. Conclusions: The review clarifies current evidence and identifies critical gaps for future research, informing clinical, educational, and preventive interventions.

## 1. Introduction

### 1.1. The Rise in Appearance-Focused Social Media

In recent years, social media platforms have become predominantly visual environments in which the sharing and evaluation of images play a central role in users’ experiences. Apps such as Instagram, TikTok, and Snapchat are particularly widespread among adolescents and young adults, who rely on them daily not only to maintain interpersonal relationships but also as a space for identity construction, self-presentation, and peer interaction (Choukas-Bradley et al., 2022). In this context, while young people are more digitally connected than ever, such immersion exposes them to significant psychiatric and psychological risks that require careful monitoring (Marano et al., 2025a). Unlike traditional media, these environments are distinguished by several specific features—including user-generated content and numerical indicators of social approval, such as likes, comments, and follower counts—which alter how girls encounter, evaluate, and internalize aesthetic ideals (Choukas-Bradley et al., 2022; Cohen et al., 2017).

A growing body of research has moved beyond aggregate indicators of social media use to examine specific patterns of engagement, drawing a key distinction between general platform use and appearance-focused activities—for instance, posting or viewing photographs, or following accounts curated around physical appearance (Cohen et al., 2017). This distinction matters empirically: across studies on adolescent girls and young women, overall time spent on social networking sites does not show a consistent relationship with body image concerns, whereas engagement with appearance-related features, such as photo-based activities on Facebook or following appearance-oriented profiles on Instagram, is reliably linked to thin-ideal internalization, body surveillance, and drive for thinness (Cohen et al., 2017; Dumas & Desroches, 2019). Recent reviews further indicate that highly visual platforms such as Instagram appear more consistently associated with body image concerns than predominantly textual ones, and that specific appearance-related activities, including taking and editing selfies, may be especially detrimental, while body positive content emerges so far as the most promising form of potentially protective material (Vandenbosch et al., 2022). More broadly, social networking sites have become a central channel through which thin and fit body ideals circulate among young women, with influencer-driven content reinforcing appearance standards that most users cannot realistically attain (Aparicio-Martinez et al., 2019).

The rise in appearance-focused social media has been accompanied by the proliferation of distinct content subcultures, ranging from explicitly harmful pro-anorexia (“Pro-Ana”) communities to ostensibly health-oriented “fitspiration” pages. Although these two types of content differ markedly in their stated purpose, experimental evidence suggests that both can adversely affect young women, albeit through divergent pathways: exposure to Pro-Ana material has been linked primarily to heightened negative and anxious affect, whereas fitspiration content appears to elicit appearance-related social comparison processes (Jennings et al., 2021). In response to this idealized visual environment, counter-movements such as body positivity (#bopo) have gained traction online, seeking to challenge dominant beauty norms by foregrounding a wider range of bodies and promoting acceptance and inclusivity (Rodgers et al., 2022). These trends underscore how central appearance-related content has become within contemporary digital environments and point to the need for a more systematic understanding of its psychological consequences for the populations most exposed to it.

### 1.2. Body Image and Psychological Development in Adolescence

Adolescence constitutes a developmental stage marked by profound restructuring at the biological, cognitive, and relational levels, within which the perception of one’s own body takes on decisive weight in the construction of identity and in psychological wellbeing (Smolak, 2004). Scholars converge in describing body image as a construct articulated across multiple layers (perceptual, affective, cognitive, evaluative, and behavioral investment) which concern, respectively, how a person sees their body, how they feel about it, how they judge it, how satisfied they are with it, and how much importance they assign to it in their everyday choices (Smolak, 2004; Thompson et al., 1999). This investment reflects how the body can become a metaphorical “battlefield” where identity conflicts and psychosomatic expressions are played out, particularly throughout childhood and adolescence (Marano et al., 2025b). At this stage, the convergence of pubertal transformations, heightened self-awareness, and the growing weight that peers acquire as a reference point makes adolescents particularly vulnerable to absorbing culturally dominant aesthetic models and to continuously measuring themselves against them (Choukas-Bradley et al., 2022; Smolak, 2004).

A substantial body of research indicates that body dissatisfaction commonly arises in early adolescence and, by the late elementary and middle-school years, tends to show meaningful stability over time, with longitudinal studies linking adolescent body dissatisfaction to the later onset of eating problems, eating disorders, and depressive symptoms (Thompson et al., 1999). A foundational account of these processes is offered by the Tripartite Influence Model (Donovan et al., 2020), which singles out three sociocultural agents (peers, parents, and the media) as the main channels through which culturally dominant appearance ideals reach young people. In this regard, it is essential to recognize that parental influence is not limited to the maternal figure, as paternal mental health and the presence of perinatal depressive symptoms can also profoundly shape the early developmental environment (Mazza et al., 2022). Within this framework, exposure to such influences is thought to translate into body dissatisfaction and disordered eating not directly, but rather through two intervening processes: the tendency to compare one’s own appearance with that of others, and the personal endorsement of culturally prescribed beauty standards (Thompson et al., 1999). More recent empirical work conducted with adolescent samples has lent further support to the explanatory weight of these mediating pathways (Rodgers et al., 2020).

Beyond its direct association with body dissatisfaction, adolescence is also marked by the emergence of cognitive structures related to physical appearance, which act as a filter in the processing of sociocultural messages about the body (Smolak, 2004). According to Smolak (2004), although these schemas begin to take shape in childhood, they remain in a relatively immature form during early adolescence, and this gradual process of construction may help explain why younger children appear comparatively less permeable to media-related pressures than older adolescents. At the same time, it is during adolescence that gender differences in body-related concerns become most pronounced: girls tend to report higher levels of body dissatisfaction, greater investment in appearance, and exposure to more consistent sociocultural messages conveyed across media, peers, and parents (Smolak, 2004). The centrality of peer feedback and identity formation processes that characterize this developmental stage (Choukas-Bradley et al., 2022), together with the pervasiveness of digital environments heavily oriented toward physical appearance, make adolescence a particularly sensitive window for the emergence of body image difficulties, which in turn represent a well-documented risk factor for depressive symptoms and disordered eating (Choukas-Bradley et al., 2022; Smolak, 2004). Furthermore, developmental pressures and difficulties in emotional regulation during this stage may also manifest through externalizing trajectories, including antisocial behaviors driven by complex psychological mechanisms (Mazza et al., 2025).

### 1.3. Gender-Specific Vulnerability

While body image concerns can affect individuals of any gender, the empirical literature consistently shows that adolescent girls and young women are disproportionately exposed to body dissatisfaction, appearance-related distress, and the psychological burden associated with internalizing idealized representations of the body (Fredrickson & Roberts, 1997; Smolak, 2004). A theoretical account of this gendered vulnerability is offered by Objectification Theory (Fredrickson & Roberts, 1997), according to which living in a sociocultural context that treats the female body as an object of evaluation leads many girls and women to gradually take on a third-person, externalized view of their own physical appearance, a process the authors describe as self-objectification. The chronic appearance-monitoring that follows from this internalized outsider perspective has been linked to a constellation of psychological consequences: more frequent experiences of body-related shame and appearance-related anxiety, fewer occasions of full absorption in rewarding activities, and a reduced ability to detect and interpret internal bodily cues. Over time, the accumulation of these experiences is thought to contribute to several forms of psychological distress that show a marked female prevalence, notably depressive symptomatology, difficulties in sexual functioning, and disordered eating patterns (Fredrickson & Roberts, 1997).

The mechanisms underlying this heightened vulnerability are multiple and interrelated. Building on Festinger’s (1954) Social Comparison Theory, which posits that individuals evaluate themselves by measuring their attributes against those of others, contemporary research has consistently identified appearance-based social comparison as a key process linking media exposure to body image concerns in girls and women (Scully et al., 2023; Thompson et al., 1999). Adolescent girls, in particular, tend to engage in upward comparisons with idealized female targets, evaluating their own appearance as less favorable than that of others (Scully et al., 2023). Such comparisons have been empirically linked to greater body dissatisfaction and lower self-esteem (McComb & Mills, 2021; Scully et al., 2023), as well as to elevated drive for thinness and broader eating disturbance (Thompson et al., 1999). Recent evidence indicates that unfavorable comparisons with close friends, rather than with celebrities or more distal peers, are most strongly associated with poorer body image appraisals among adolescent girls (Scully et al., 2023), highlighting the role of proximal social environments in shaping appearance-related self-evaluations. Beyond correlational evidence, a recent systematic review of 43 experimental studies has provided causal support for the impact of viewing idealized images on social networking sites on body dissatisfaction in young women, identifying state appearance comparison as a significant mediator and trait appearance comparison as a moderator of this effect (Fioravanti et al., 2022).

Beyond comparison processes, the internalization of culturally promoted appearance standards represents a second pathway linking sociocultural pressures to body-related distress. Through repeated exposure to curated and digitally edited depictions of slender female bodies on social networking platforms, adolescent girls and young women come to endorse these standards as personal benchmarks against which their own bodies are evaluated (Aparicio-Martinez et al., 2019; Cohen et al., 2017). Empirical work has consistently linked stronger endorsement of such ideals to greater body dissatisfaction, drive for thinness, and disordered eating attitudes (Cohen et al., 2017; Rodgers et al., 2020). Rather than acting in isolation, internalization appears to function alongside self-objectification and upward appearance comparison, with these mechanisms reinforcing one another to heighten vulnerability to negative body-image outcomes in adolescent and emerging-adult women (Choukas-Bradley et al., 2022; Fredrickson & Roberts, 1997).

The convergence of these processes appears especially pronounced during adolescence, a developmental stage in which peer evaluation, identity exploration, and sensitivity to social feedback become particularly salient (Choukas-Bradley et al., 2022). The “perfect storm” framework articulated by Choukas-Bradley et al. (2022) accounts for this convergence by bringing together developmental processes, broader sociocultural pressures, and gender-related dynamics to explain why adolescent girls are disproportionately vulnerable in contemporary appearance-focused digital environments. Within this account, the specific affordances of social media platforms operate alongside the heightened importance of peer feedback in adolescence and alongside longstanding cultural pressures that center women’s physical appearance, producing conditions in which body image concerns are likely to intensify and, in turn, contribute to depressive symptoms and disordered eating (Choukas-Bradley et al., 2022; Rodgers et al., 2020).

### 1.4. Heterogeneity of the Evidence and Rationale for the Review

Despite a rapidly growing body of research examining how social media use relates to body image and psychological wellbeing in adolescent girls and young women, the available evidence remains notably heterogeneous across study designs, target populations, platforms considered, and outcome measures (Dumas & Desroches, 2019; Rodgers et al., 2022). Recent syntheses have begun to map portions of this literature: Dane and Bhatia (2023) systematically reviewed the relationship between social media use, body image, and eating disorders in young people aged 10–24 years of any gender, drawing on 50 studies from 17 countries published between January 2016 and July 2021, and adopted an explicit global public health framework to discuss social media as a risk factor for clinical and subclinical eating pathology (Dane & Bhatia, 2023). Fioravanti et al. (2022) synthesized 43 experimental investigations of idealized image exposure on social networking sites, providing causal evidence for the role of state appearance comparison as a mediating mechanism (Fioravanti et al., 2022). These important contributions, however, have either centered on eating disorders as the principal endpoint within mixed-gender populations and a relatively narrow temporal window, or have been confined to experimental designs, leaving the broader, more heterogeneous, and more recent body of evidence on body image, self-esteem, and psychological wellbeing in adolescent girls and young women only partially mapped. In particular, the rapid growth of video-based platforms such as TikTok, the proliferation of body positive content, and the marked acceleration of social media use during and after the COVID-19 pandemic have substantially reshaped the empirical landscape since 2021, warranting an updated, gender-specific, and developmentally informed synthesis of the available evidence. Alongside the surge in social media use, the pandemic period fostered other dysfunctional coping mechanisms, such as increased alcohol consumption and its associated systemic complications (Marano et al., 2022). A particularly salient source of variability concerns the operationalization of body image itself: a wide range of constructs (spanning body satisfaction, body appreciation, self-objectification, body surveillance, drive for thinness, and internalization of appearance ideals, among others) has been assessed through an equally varied set of psychometric instruments, often with different tools used to capture the same underlying dimension (Dumas & Desroches, 2019). On the content side, social media exposure has been examined in relation to idealized imagery, fitspiration, body positive posts, peer feedback, and self-photo activities, yet variations in these elements have rarely been manipulated within a single systematic program of research, leaving the relative contribution of specific content features unclear (Rodgers et al., 2022). The result is an evidence base in which findings are difficult to integrate across platforms, content types, and methodologies, and in which moderating factors such as appearance comparison tendency, thin-ideal internalization, social media literacy, and appearance schematicity appear to shape outcomes in ways that are still only partially understood (Dumas & Desroches, 2019; Rodgers et al., 2022).

A first source of heterogeneity concerns the way social media exposure itself has been conceptualized and operationalized. Earlier work tended to rely on broad indicators such as total time spent on social networking sites or general frequency of use (Cohen et al., 2017; Dumas & Desroches, 2019), but more recent findings indicate that such global metrics may obscure meaningful effects, since the qualitative nature of engagement, and especially exposure to appearance-focused content, appears to be a more consistent correlate of adverse body image outcomes than overall use (Cohen et al., 2017; Dumas & Desroches, 2019). Even within appearance-related material, however, the distinctions between content categories are far from sharp: pro-anorexia and fitspiration imagery, for instance, share enough thematic overlap that the line between them has been described as blurred, despite evidence that the two elicit divergent cognitive and emotional responses, with fitspiration content tending to trigger social comparison processes and pro-anorexia content evoking more pronounced negative and anxious affect (Jennings et al., 2021). Body positive content adds a further layer of complexity, as it is itself heterogeneous in imagery, messaging, and underlying philosophy, with some posts paradoxically reproducing the very appearance ideals they purport to challenge (Rodgers et al., 2022). A more systematic mapping of how these distinct, and at times overlapping, types of content relate to body image and psychological outcomes therefore remains an open priority for the field (Cohen et al., 2017; Rodgers et al., 2022).

A second source of heterogeneity concerns the psychological mechanisms proposed to explain the relationship between social media exposure and body image-related outcomes. Processes such as appearance-based social comparison, thin-ideal internalization, and self-objectification have consistently been implicated within sociocultural and biopsychosocial frameworks (Fredrickson & Roberts, 1997; Rodgers et al., 2020; Scully et al., 2023; Thompson et al., 1999). Appearance-based social comparison has been identified as a key mediational process linking appearance-related experiences to body dissatisfaction and eating-related disturbances (Thompson et al., 1999), while appearance-focused social media activity has been associated with body dissatisfaction through increased upward social comparison and greater internalization of appearance ideals (Scully et al., 2023). Nevertheless, substantial variability persists regarding which psychological mechanisms are assessed and how they are integrated into broader explanatory models across studies. Furthermore, individual vulnerability factors, including physical appearance perfectionism (McComb & Mills, 2021), self-esteem and body dissatisfaction (Aparicio-Martinez et al., 2019), as well as appearance-focused patterns of social media engagement (Cohen et al., 2017), have been investigated inconsistently, limiting efforts to identify individuals who may be particularly susceptible to adverse psychological outcomes. The need for a more refined conceptualization of appearance-focused social media engagement is also supported by measurement-focused studies that have developed instruments specifically targeting image-based activity and appearance comparison on Instagram. Di Gesto et al. (2020) introduced validated measures of Instagram image-related activities and Instagram appearance comparison, highlighting that appearance-focused engagement is not reducible to general platform use but includes specific behaviors such as viewing, posting, editing, and comparing appearance-related images. More recently, the construct of appearance-related social media consciousness has further broadened the field by capturing the extent to which individuals think about how their own appearance will be evaluated in social media contexts (Di Gesto et al., 2025).

All these sources of heterogeneity highlight the need for a structured mapping of the evidence base capable of clarifying conceptual boundaries, integrating risk and protective factors, and identifying directions for future research. These contributions support the present review’s emphasis on distinguishing between exposure, active self-presentation, comparison processes, and appearance-related cognitive preoccupation.

### 1.5. Objectives

Considering the conceptual fragmentation, methodological heterogeneity, and uneven integration of risk and vulnerability factors that characterize the existing literature, the present scoping review aims to systematically map the extent, nature, and characteristics of the available evidence on the relationship between appearance-focused social media use, body image, self-esteem, and psychological wellbeing in adolescent girls and young women.

More specifically, the review seeks to map the range of appearance-focused social media content and activities that have been examined in this body of research (including exposure to idealized imagery, photo-based engagement, fitspiration and pro-anorexia content, body positive material, and peer feedback) and to clarify how these distinct, and at times overlapping, content categories have been operationalized across studies. In parallel, the review aims to chart the breadth of body image, self-esteem, and psychological wellbeing outcomes that have been investigated, spanning constructs such as body dissatisfaction, drive for thinness, self-objectification, body surveillance, depressive symptomatology, anxiety, and disordered eating attitudes and behaviors, with attention to the variety of psychometric instruments employed to capture these dimensions.

A further objective is to examine the psychological mechanisms most frequently proposed and empirically tested as mediators of the relationship between appearance-focused social media use and adverse outcomes, with particular attention to appearance-based social comparison, internalization of appearance ideals, and self-objectification, as well as to the extent to which these processes are integrated within broader sociocultural and biopsychosocial frameworks. The review also intends to map moderating, vulnerability, and protective factors reported in the literature, including individual-level variables (e.g., self-esteem, body dissatisfaction, physical appearance perfectionism), patterns of appearance-focused social media engagement, and emerging constructs such as social media literacy, appearance schematicity, and engagement with body positive content.

Finally, the review aims to describe the methodological characteristics of the existing evidence base (including study designs, populations examined, measurement tools, and theoretical frameworks adopted) and, on this basis, to identify conceptual and empirical gaps that may inform future research priorities and the development of evidence-based clinical, educational, and preventive interventions targeting body image and psychological wellbeing in adolescent girls and young women.

The novelty of the present review lies in its attempt to map appearance-focused social media engagement as a multidimensional construct rather than as a single exposure variable. Unlike previous reviews that have focused primarily on eating disorder outcomes, mixed-gender youth samples, or experimental exposure to idealized images (Dane & Bhatia, 2023; Fioravanti et al., 2022; Vandenbosch et al., 2022), this scoping review adopts a gender-specific and developmentally informed lens and includes a broader range of outcomes, including body dissatisfaction, positive body image, self-esteem, self-objectification, affective wellbeing, anxiety, depressive symptoms, and disordered eating attitudes. A scoping review methodology was chosen because the literature is conceptually and methodologically heterogeneous, with substantial variation in platforms, content types, engagement behaviors, theoretical mechanisms, outcomes, and instruments. The purpose was therefore not to estimate a pooled effect size, but to clarify the breadth of the field, identify how key constructs have been operationalized, map mechanisms and moderators, and highlight conceptual and empirical gaps requiring future systematic or meta-analytic investigation (Arksey & O’Malley, 2005; Levac et al., 2010; Peters et al., 2020).

By providing a comprehensive and structured mapping of the available evidence, this scoping review seeks to contribute to a clearer conceptualization of the relationship between appearance-focused social media use and body-related psychological outcomes, and to support the design of future research and intervention efforts in this rapidly evolving field.

## 2. Theoretical Frameworks

The relationship between appearance-focused social media use and body image in adolescent girls and young women has been examined through several complementary theoretical perspectives, three of which provide the conceptual backbone of the present scoping review. Social Comparison Theory (Festinger, 1954) posits a fundamental human drive to evaluate one’s own attributes by comparison with others; when applied to physical appearance, such comparisons typically take an upward direction toward more attractive targets and have been consistently associated with body dissatisfaction, lowered self-esteem, and disordered eating attitudes (McComb & Mills, 2021; Scully et al., 2023; Thompson et al., 1999). Objectification Theory (Fredrickson & Roberts, 1997) further situates these dynamics within a broader sociocultural analysis, proposing that exposure to a culture that sexually objectifies the female body leads many girls and women to internalize an external observer’s perspective on themselves—a process termed self-objectification, behaviorally expressed as habitual body surveillance and emotionally as body shame (McKinley & Hyde, 1996). Recent extensions have argued that visual social media platforms, by foregrounding self-photographs and quantifiable peer feedback, may intensify these processes through what has been described as online self-objectification (Vandenbosch & Eggermont, 2012). Finally, the internalization of culturally promoted appearance ideals, that is, the personal endorsement of such standards as guiding values for self-evaluation, has been identified as one of the most robust proximal risk factors for body dissatisfaction and eating pathology (Schaefer et al., 2015; Thompson & Stice, 2001).

These three constructs (appearance comparison, self-objectification, and internalization of appearance ideals) converge within broader sociocultural models of body image, most notably the Tripartite Influence Model (Thompson et al., 1999), which identifies peers, family, and the media as the principal channels through which appearance ideals are transmitted, and more recent biopsychosocial extensions that integrate these mechanisms with individual vulnerability factors in the specific context of social media use (Rodgers et al., 2020). Building on these foundations, the perfect storm framework articulated by Choukas-Bradley et al. (2022) offers an integrative developmental–sociocultural account in which the affordances of social media platforms intersect with the heightened salience of peer relationships in adolescence and with longstanding sociocultural pressures centered on female appearance, positioning body image concerns as a key mechanism linking adolescent girls’ social media use to depressive symptoms and disordered eating (Choukas-Bradley et al., 2022). Furthermore, these psychiatric outcomes may carry broader systemic implications, as chronic psychological distress is increasingly recognized to interact with physiological processes, potentially influencing the long-term clinical outlook and overall physical wellbeing (Mazza et al., 2024). These perspectives provide the conceptual lens through which the present scoping review interprets the available evidence.

**Figure 1:**
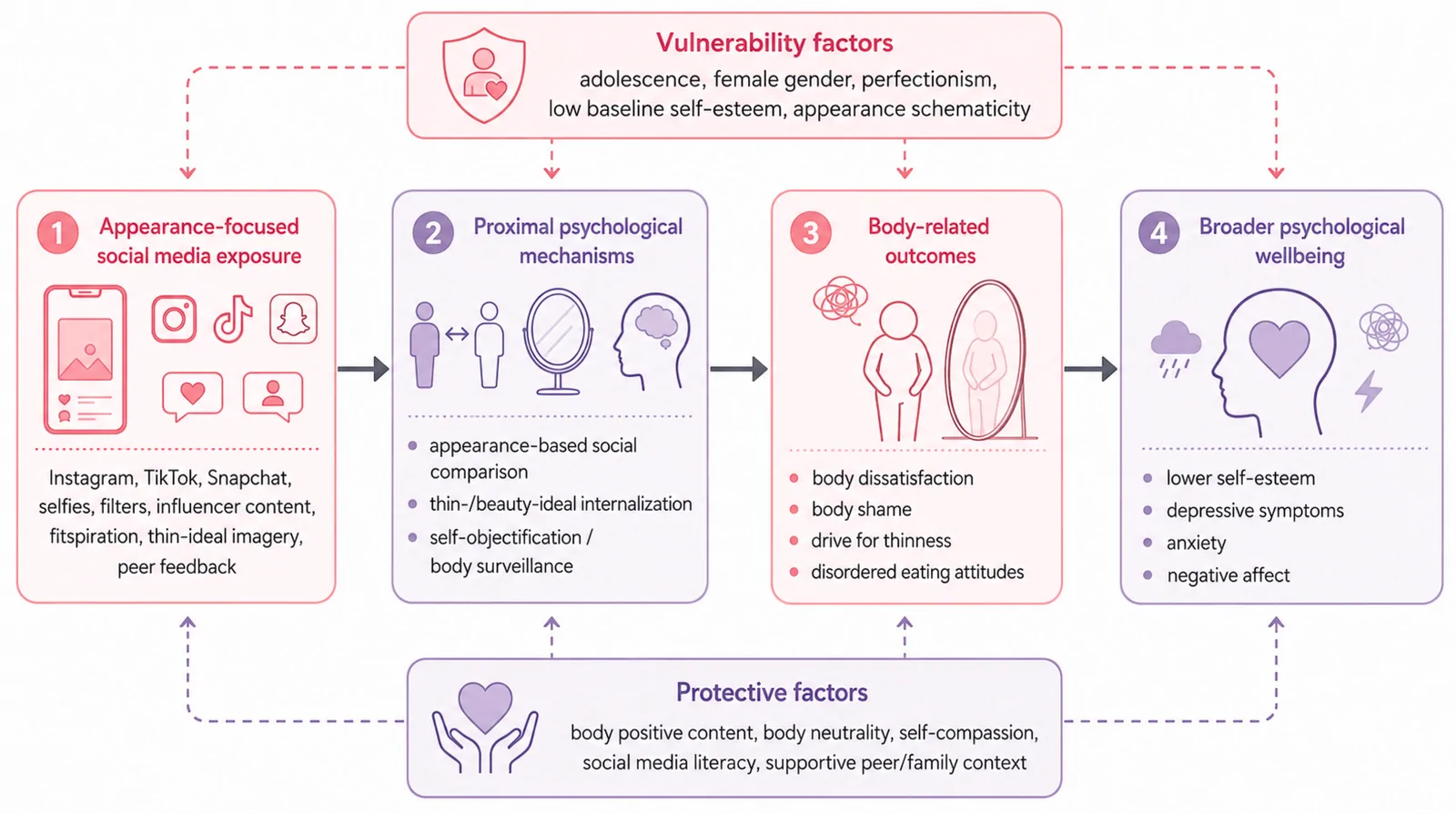
Conceptual pathway linking appearance-focused social media exposure to body image and psychological wellbeing outcomes. Note: The model integrates social comparison, internalization of appearance ideals, and self-objectification as core mechanisms through which appearance-focused digital environments may contribute to body dissatisfaction, body shame, drive for thinness, disordered eating attitudes, reduced self-esteem, depressive symptoms, anxiety, and negative affect. Vulnerability and protective factors are shown as contextual modifiers of these pathways.

To visually integrate the theoretical frameworks underpinning this review, Figure 1 summarizes the proposed pathway through which appearance-focused social media exposure may influence body image and psychological wellbeing in adolescent girls and young women. The model highlights three recurrent mediating processes, appearance-based social comparison, internalization of appearance ideals, and self-objectification, while also situating these pathways within vulnerability and protective factors identified across the literature.

## 3. Materials and Methods

### 3.1. Study Design and Protocol Registration

The present scoping review was conducted in accordance with the methodological framework originally proposed by Arksey and O’Malley (2005) and subsequently refined by Levac et al. (2010), and was further informed by the updated methodological guidance provided by the Joanna Briggs Institute (Arksey & O’Malley, 2005; Levac et al., 2010; Peters et al., 2020). Reporting follows the Preferred Reporting Items for Systematic Reviews and Meta-Analyses extension for Scoping Reviews (PRISMA-ScR) checklist (Tricco et al., 2018).

The review protocol was registered on the Open Science Framework (OSF; retrieved from: https://osf.io/73hfd/overview; accessed on 8 June 2026). As the registration was completed after the searches had been conducted, it constitutes a retrospective registration; the protocol, eligibility criteria, and charting framework were nonetheless defined prior to data extraction and were not altered based on the findings. 

### 3.2. Eligibility Criteria

Eligibility criteria were defined according to the Population-Concept-Context (PCC) framework recommended by the JBI methodology for scoping reviews (Peters et al., 2020) and were iteratively refined during the screening process, consistent with the methodological guidance provided by Levac et al. (2010), to enhance the focus, homogeneity, and feasibility of the synthesis. The final eligibility criteria are reported below.

With respect to population, eligible studies were those examining exclusively female samples of participants aged between 10 and 30 years, with a minimum sample size of *n* ≥ 50. The decision to include females aged 10–30 years was based on both developmental and field-specific considerations. The lower boundary of 10 years was chosen to capture the onset of early adolescence, when pubertal changes, peer comparison, and appearance-related self-evaluation begin to intensify (Choukas-Bradley et al., 2022; Smolak, 2004). The upper boundary of 30 years was selected to include emerging adulthood and young adulthood, which represent developmental periods in which identity consolidation, intimate relationships, and intensive use of visual social media platforms remain highly salient for body image and self-esteem (Choukas-Bradley et al., 2022; Vandenbosch et al., 2022). It is important to recognize that this range is developmentally broad and does not imply homogeneity across ages. Therefore, throughout the synthesis, we distinguished between younger adolescent samples, broadly corresponding to participants aged 10–17 years, and emerging-adult/young-women samples, broadly corresponding to participants aged 18–30 years. This distinction was used descriptively to highlight developmental imbalances in the evidence base rather than to impose a formal subgroup comparison, which was not feasible given the limited number of studies focusing exclusively on younger adolescents. With respect to concept, eligible studies investigated the relationship between appearance-focused social media use, including, but not limited to, exposure to image-based platforms (e.g., Instagram, TikTok, Snapchat), photo-related activities (taking, editing, posting, viewing), engagement with appearance-oriented content (e.g., fitspiration, thinspiration, body positive content), and exposure to influencer or peer-generated appearance content, and at least one of the following outcomes: body image, self-esteem, or psychological wellbeing, operationalized through constructs such as body dissatisfaction, body appreciation, self-objectification, body surveillance, drive for thinness, internalization of appearance ideals, depressive symptoms, anxiety, or disordered eating attitudes and behaviors. To preserve the directionality of the hypothesized relationship, social media use was required to be measured as the exposure or independent variable, rather than as the outcome. With respect to context, no restrictions were applied: studies were eligible regardless of geographical setting, recruitment context (community, school-based, clinical, or online), or cultural background.

In terms of study design, all primary empirical studies adopting quantitative, qualitative, or mixed-methods approaches were considered eligible, including cross-sectional, longitudinal, and experimental designs. Existing systematic and scoping reviews were not included as primary sources but were screened to identify additional relevant studies through reference checking. Editorials, commentaries, conference abstracts, dissertations, book chapters, and grey literature were excluded. Studies evaluating the effectiveness of interventions, including school-based prevention programs, social media literacy training, and clinical or therapeutic interventions, were also excluded, as the present review focuses on mapping correlational, observational, and experimental evidence on the relationship between appearance-focused social media use and body-related psychological outcomes, rather than on evaluating the efficacy of interventions targeting these outcomes. Only peer-reviewed articles published in English between January 2010 (coinciding with the launch of Instagram and the consolidation of image-based social media platforms) and May 2026 were considered.

The restriction to female samples was chosen because the review focused on the population most consistently identified in the literature as being at elevated risk for appearance-related body dissatisfaction, self-objectification, and internalization of culturally prescribed appearance ideals (Choukas-Bradley et al., 2022; Rodgers et al., 2020; Scully et al., 2023). The age range of 10–30 years was selected to capture the developmental continuum from early adolescence to emerging adulthood and young adulthood; however, this range was not treated as developmentally homogeneous, and age-related imbalances in the evidence base were explicitly mapped. Intervention studies were excluded because the aim of the review was not to evaluate the effectiveness of prevention or treatment programs, but to map naturally occurring and experimentally induced associations between appearance-focused social media engagement and body-related psychological outcomes. Studies in which social media use was treated as the outcome variable were excluded to preserve the conceptual direction of the review question, which focused on social media exposure or engagement as a potential correlate, predictor, or experimental stimulus in relation to body image, self-esteem, and psychological wellbeing. These eligibility restrictions improve conceptual focus but also limit generalizability, particularly to males, gender-diverse populations, clinical samples, and intervention contexts.

### 3.3. Search Strategy

A comprehensive search strategy was developed in consultation among the authors and was implemented across three electronic bibliographic databases: PubMed, Scopus, and Web of Science. The search combined four thematic blocks connected by the Boolean operator AND: (i) social media platforms (e.g., “social media”, “social networking”, Instagram, TikTok, “image-based platform*”); (ii) body image and related constructs (e.g., “body image”, “body dissatisfaction”, “self-objectification”, “body surveillance”, “self-esteem”); (iii) the target population (“adolescen*”, “young women”, “girl*”); and (iv) appearance-focused framing (“appearance-focused”, “appearance-related”, “appearance comparison”). Search syntax was adapted to each database, including the use of MeSH terms in PubMed. The full search strings are reported in Table S1.

To ensure comprehensive coverage of the available evidence, the database search was supplemented by manual screening of the reference lists of all included studies and of relevant systematic and scoping reviews identified during the screening process (snowballing technique). The literature search was conducted between 1 April and 15 May 2026.

### 3.4. Study Selection

**Figure 2:**
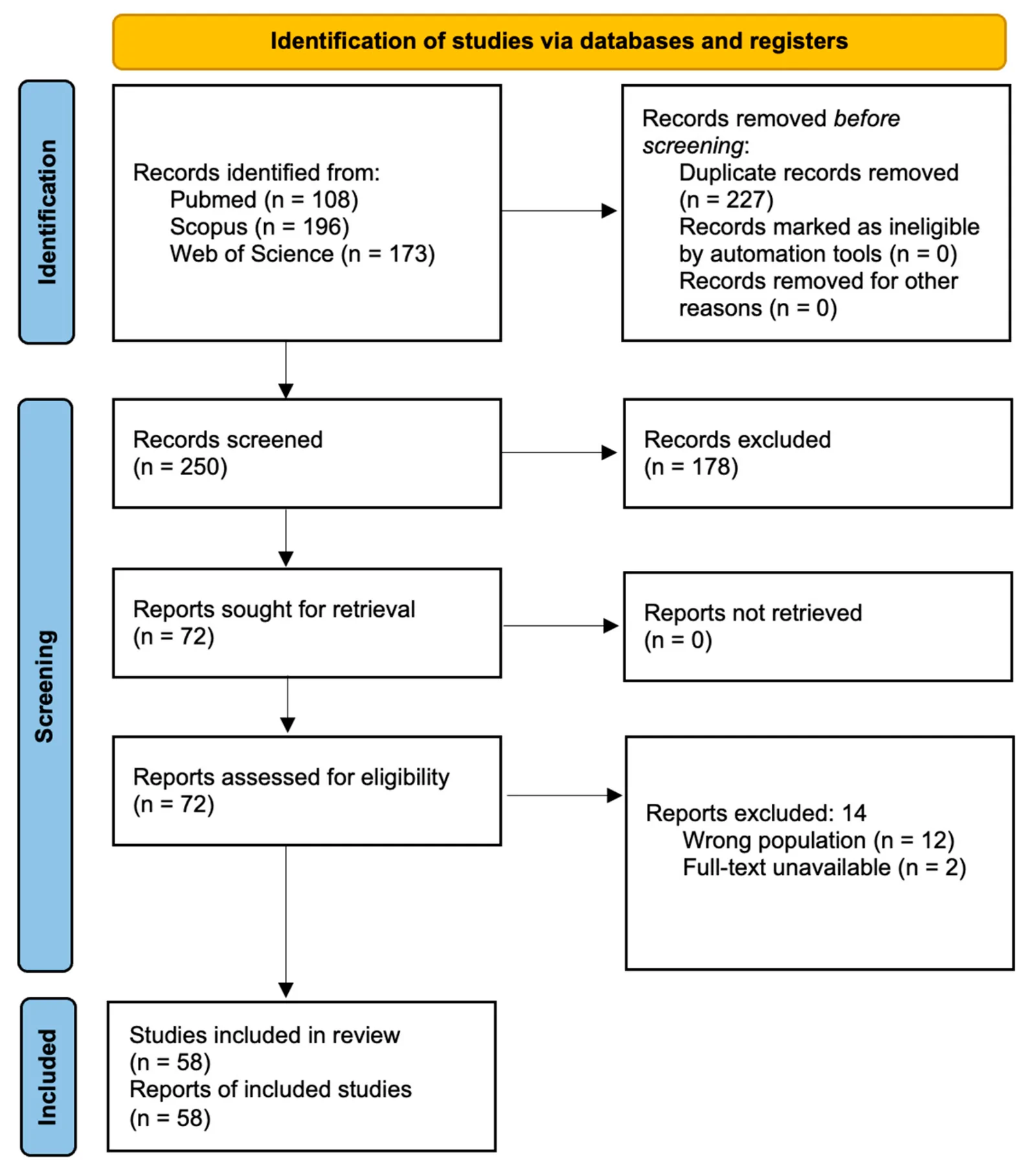
PRISMA-ScR flow diagram.

All records retrieved through the database searches were exported and consolidated in Mendeley Reference Manager (Mendeley Ltd., Elsevier), where duplicate records were identified and removed through a combination of automatic detection and manual verification. The screening process was conducted in two sequential stages. In the first stage, two reviewers independently screened the titles and abstracts of all retrieved records against the predefined eligibility criteria. Records deemed potentially eligible by at least one reviewer were retained for the second stage. In the second stage, the full texts of the retained records were retrieved and independently assessed for eligibility by the same two reviewers. Disagreements at either stage were resolved through discussion, and a third reviewer was consulted in cases of persistent disagreement. Inter-rater agreement was assessed using Cohen’s kappa coefficient, with values above 0.80 considered indicative of substantial agreement (McHugh, 2012). The study selection process is summarized in a PRISMA-ScR flow diagram (Figure 2), which reports the number of records identified, screened, assessed for eligibility, and ultimately included in the review, together with the reasons for exclusion at the full-text stage.

### 3.5. Data Charting and Extraction

A standardized data charting form was developed a priori by the authors, based on the review objectives and the recommendations provided by the JBI methodology (Peters et al., 2020). The form was piloted on a random subset of five included studies to assess clarity, comprehensiveness, and consistency, and was subsequently refined through team discussion before being applied to the full set of included records. Data were extracted independently by one reviewer and verified by a second reviewer; any discrepancies were resolved through discussion.

The following information was charted from each included study: bibliographic details (first author, year of publication, country, journal); study design and methodological approach; sample characteristics (size, age range and mean age, gender composition, recruitment setting); social media platforms and types of appearance-focused content or activities examined; body image, self-esteem, and psychological wellbeing constructs assessed, together with the corresponding measurement instruments; theoretical framework explicitly adopted, where reported; main findings relevant to the review questions; mediating, moderating, and protective variables identified; limitations and future research directions reported by the authors. In line with the methodological guidance for scoping reviews (Peters et al., 2020; Tricco et al., 2018), no formal critical appraisal of methodological quality was undertaken, given that the aim of the review is to map the breadth and characteristics of the available evidence rather than to evaluate the strength of individual findings.

### 3.6. Data Synthesis

Consistent with the descriptive and exploratory nature of scoping review methodology, data were synthesized using a narrative approach, complemented by tabular and graphical summaries (Peters et al., 2020; Tricco et al., 2018). Quantitative aggregation through meta-analysis was not pursued, in line with the mapping aims of the review. Findings were organized around the key thematic domains identified a priori from the review objectives: types of appearance-focused social media content and activities examined; body image, self-esteem, and psychological wellbeing outcomes investigated, including the measurement instruments employed; psychological mechanisms tested as mediators of the relationship between appearance-focused social media use and outcomes (e.g., appearance-based social comparison, internalization of appearance ideals, self-objectification); moderating, vulnerability, and protective factors identified, including individual-level variables and emerging constructs such as social media literacy and engagement with body positive content; methodological characteristics of the evidence base, including study designs, populations, theoretical frameworks, and geographical distribution. Frequencies of key variables (e.g., platforms examined, outcomes assessed, theoretical frameworks adopted) were summarized in descriptive tables to facilitate the identification of recurring patterns and gaps. The thematic synthesis was discussed iteratively among the authors to ensure consistency of categorization and interpretation.

## 4. Results

### 4.1. Search Results and Study Selection

The database searches retrieved 477 records overall (108 from PubMed, 196 from Scopus, and 173 from Web of Science) between April and May 2026. Removal of 227 duplicates in Mendeley left 250 unique records, which were screened on title and abstract; 178 were excluded at this stage. Two reviewers then independently assessed the full texts of the remaining 72 articles, excluding 14 further records: 12 because the sample fell outside the population criteria (mixed-gender or male-only samples, or female samples outside the 10–30 age range) and 2 because the full text could not be retrieved. Inter-rater agreement was substantial throughout (Cohen’s κ > 0.80). Fifty-eight studies entered the final synthesis. Table 1 summarizes the flow.

### 4.2. Characteristics of Included Studies

The 58 studies included in the review are summarised in Table 1 and reported in full in Supplementary Tables S2 and S3 (Anixiadis et al., 2019; Becker et al., 2025; Belmonte et al., 2024; Brown & Tiggemann, 2016; Chang et al., 2019; Chansiri et al., 2022; Choukas-Bradley et al., 2019; Cohen et al., 2017; Cohen & Blaszczynski, 2015; Dajches et al., 2025; Daniels & Robnett, 2021; Engeln et al., 2020; Etherson et al., 2022; Fardouly et al., 2015b, 2015a, 2017, 2018; Fardouly & Holland, 2018; Fardouly & Vartanian, 2015; Ferdousi et al., 2023; Fioravanti et al., 2023, 2024; Flynn & Newman, 2023; Galway & Gammage, 2025; Hai & Yang, 2022; Hogue & Mills, 2019; Kim, 2018; Kowal et al., 2026; Lee, 2025; Lee & Lee, 2021; Livingston et al., 2020; Markey & Daniels, 2022; McComb & Mills, 2021; Meier & Gray, 2014; Patel et al., 2025; Prichard et al., 2023; Pryde & Prichard, 2022; Roberts et al., 2022; Roorda et al., 2026; Rousseau, 2024; Samson & Zaitsoff, 2023; Scully et al., 2023; Seekis et al., 2020, 2021; Seekis & Kennedy, 2023; Seekis & Lawrence, 2023; Slater & Tiggemann, 2015; Song et al., 2025; Tiggemann et al., 2018; Tiggemann & Barbato, 2018; Tiggemann & Zaccardo, 2015; Vendemia & Fox, 2024; Walker et al., 2015; Yang et al., 2020; Yue & Tang, 2024; Zhong, 2022; Zhou et al., 2024; Žulec Ivanković et al., 2024). The key characteristics of these studies are presented below; full study-level detail is reported in Supplementary Table S2. Publication dates span the period 2014–2026, but the geographical distribution is anything but balanced. Seventeen studies came from Australia (e.g., Cohen & Blaszczynski, 2015; Slater & Tiggemann, 2015; Tiggemann & Zaccardo, 2015; see Supplementary Table S2) and 13 from the United States (e.g., Choukas-Bradley et al., 2019; Fardouly & Holland, 2018; Meier & Gray, 2014; see Supplementary Table S2). Together, these two countries account for over half of the entire evidence base. China is represented by 5 studies (Hai & Yang, 2022; Song et al., 2025; Yue & Tang, 2024; Zhong, 2022; Zhou et al., 2024), while Canada (Hogue & Mills, 2019; McComb & Mills, 2021; Roorda et al., 2026; Samson & Zaitsoff, 2023) and the United Kingdom (Etherson et al., 2022; Fardouly et al., 2015a, 2015b; Flynn & Newman, 2023) contribute 4 each. South Korea contributes 3 studies (Kim, 2018; Lee, 2025; Lee & Lee, 2021), Italy and Singapore 2 each (Fioravanti et al., 2023, 2024; and Chang et al., 2019; Yang et al., 2020, respectively) and Poland, Croatia, Ireland, and Belgium 1 each (Kowal et al., 2026; Rousseau, 2024; Scully et al., 2023; Žulec Ivanković et al., 2024). A further 4 investigations were multinational (Galway & Gammage, 2025; Fardouly et al., 2018; Becker et al., 2025; Anixiadis et al., 2019). Sub-Saharan Africa, the Middle East, and Latin America are entirely absent; a gap that, given the cultural specificity of beauty ideals, deserves attention in future work.

Three designs dominate the methodological landscape. Cross-sectional surveys account for half of the body of evidence (*n* = 29), combining self-report questionnaires with regression, path analysis, or structural equation modelling. Experimental designs follow closely (*n* = 25): most use between-subjects protocols in which participants are randomly assigned to view appearance-focused stimuli (thin-ideal, fitspiration, or beauty content) versus appearance-neutral controls (travel images, nature scenery, landscape paintings). Only 4 studies adopt longitudinal or intensive longitudinal methods (ecological momentary assessment (Fardouly et al., 2017), experience sampling over 28 consecutive days (Fioravanti et al., 2023), and multi-wave designs (Markey & Daniels, 2022)). This last category, although small, provides the strongest evidence available on within-person temporal dynamics.

Sample sizes range from *n* = 100 (Yang et al., 2020) to *n* = 2126 (Zhou et al., 2024), with most studies recruiting between 100 and 500 participants. The total cumulative sample exceeds 16,000 girls and women. Reported ages span 10 to 39 years, but the centre of gravity of the evidence lies in emerging adulthood: roughly two-thirds of the studies enrolled university students or community samples aged 18–30. Only 9 studies focused on younger adolescent girls aged 10–17 (e.g., Daniels & Robnett, 2021; Slater & Tiggemann, 2015; see Supplementary Table S2), a striking imbalance, given that adolescence is the developmental window in which body image concerns most typically take root. Recruitment relied heavily on undergraduate psychology participant pools (e.g., Cohen & Blaszczynski, 2015; Fardouly et al., 2017; Tiggemann & Zaccardo, 2015) and online platforms (Amazon Mechanical Turk, Prolific, Qualtrics, Wenjuanxing, Sojump). School-based recruitment appeared almost exclusively in studies of younger adolescents. One investigation (Fioravanti et al., 2024) recruited a clinical sample of women with diagnosed eating disorders alongside community controls. A particularly important developmental imbalance emerged. Only 9 of the 58 included studies focused specifically on younger adolescent girls aged 10–17 years, whereas 49 studies examined emerging adults or young women, most often university or online community samples. This imbalance is noteworthy because early and middle adolescence are theoretically central periods for the emergence of body image concerns, pubertal body-related self-consciousness, peer comparison, and sensitivity to appearance-related feedback. The current literature therefore provides a much stronger map of appearance-focused social media effects in emerging adult women than in early and mid-adolescent girls. Consequently, conclusions concerning younger adolescents should be interpreted cautiously and treated as a priority area for future research (Choukas-Bradley et al., 2022; Smolak, 2004).

Instagram is by far the most studied platform, appearing in 34 of the 58 included studies, alone or alongside others. Facebook appeared in 18 studies, typically older work or in combination with Instagram (e.g., Cohen & Blaszczynski, 2015; Meier & Gray, 2014; Fardouly & Vartanian, 2015; see Supplementary Table S2). TikTok, almost absent before 2022, has rapidly become a research focus, featuring in 8 studies published between 2022 and 2026 (e.g., Roorda et al., 2026; Galway & Gammage, 2025; Patel et al., 2025; see Supplementary Table S2). Four studies examined Chinese-specific platforms such as Sina Weibo, WeChat, and Xiaohongshu/Little Red Book (Yue & Tang, 2024; Zhong, 2022; Zhou et al., 2024; Song et al., 2025), reflecting a small but culturally distinct stream of work. Snapchat, YouTube, and unspecified general social networking use appeared less frequently, almost always alongside the platforms above (e.g., Slater & Tiggemann, 2015; Hai & Yang, 2022; Roberts et al., 2022).

The kinds of appearance-focused content and activities investigated are remarkably heterogeneous, and this heterogeneity matters because it sits at the heart of the conceptual fragmentation flagged in the Introduction. Frequently examined content categories include idealized or thin-ideal imagery (e.g., Fardouly & Holland, 2018; Cohen & Blaszczynski, 2015; Tiggemann et al., 2018; see Supplementary Table S2), fitspiration content (e.g., Tiggemann & Zaccardo, 2015; Fardouly et al., 2018; Seekis et al., 2021), peer- and celebrity-focused comparisons (e.g., Hogue & Mills, 2019; Brown & Tiggemann, 2016; Vendemia & Fox, 2024), and photo-based engagement such as posting, viewing, editing, or tagging photographs (e.g., Lee & Lee, 2021; Meier & Gray, 2014; Cohen et al., 2017). More recent investigations broaden the scope: body positive content (Fioravanti et al., 2023; Livingston et al., 2020; Rousseau, 2024; Becker et al., 2025), beauty and makeup tutorials (Yue & Tang, 2024), “What I Eat in a Day” (WIEIAD) videos on TikTok (Roorda et al., 2026; Galway & Gammage, 2025), before-and-after weight-loss transformations (Flynn & Newman, 2023; Samson & Zaitsoff, 2023), self-compassion content (Seekis & Lawrence, 2023), body neutrality content (Seekis & Lawrence, 2023), and sexualised peer imagery (Vendemia & Fox, 2024; Prichard et al., 2023) all appear in studies from the last four years. The trajectory is clear: research is moving beyond the narrow opposition “thin-ideal vs. neutral” toward a much richer taxonomy of online content that includes potentially protective material. To clarify this conceptual overlap, the appearance-focused exposures identified in the included studies can be grouped into five broad, partially overlapping categories. First, idealized appearance content included thin-ideal, beauty-focused, celebrity, influencer, and highly curated peer images (Brown & Tiggemann, 2016; Fardouly & Holland, 2018). Second, fitness-oriented appearance content included fitspiration, exercise-related imagery, and weight-loss transformation content, which often combined health, discipline, and appearance ideals (Samson & Zaitsoff, 2023; Tiggemann & Zaccardo, 2015). Third, self-presentation activities included selfie taking, photo editing, photo posting, tagging, and monitoring appearance-related feedback such as likes and comments (Cohen et al., 2017; Lee & Lee, 2021). Fourth, harmful or risk-promoting content included pro-anorexia, thinspiration, and content explicitly or implicitly reinforcing restrictive eating or extreme body ideals (Jennings et al., 2021; Patel et al., 2025). Fifth, potentially protective content included body positive, body neutrality, self-compassion, and body diverse material (Rodgers et al., 2022; Rousseau, 2024; Seekis & Lawrence, 2023). These categories were not mutually exclusive: fitspiration content may combine health-oriented messaging with thin- or muscular-ideal imagery (Jennings et al., 2021; Tiggemann & Zaccardo, 2015); body positive posts may continue to reproduce some conventional attractiveness norms (Becker et al., 2025; Rodgers et al., 2022); and peer or influencer images may function simultaneously as idealized appearance exposure and social comparison targets (Brown & Tiggemann, 2016; Hogue & Mills, 2019). This overlap explains why several studies were coded under more than one content or activity category and supports the need for future research to distinguish platform, content type, and engagement style more systematically.

Earlier influential studies also shaped the field by demonstrating that specific photo-based activities, rather than general social media use alone, are most closely associated with body image disturbance. Meier and Gray (2014) showed that Facebook photo activity was associated with body image concerns among adolescent girls, while Brown and Tiggemann (2016) demonstrated that exposure to attractive celebrity and peer images on Instagram adversely affected body image in young women. Together with more recent measurement work on Instagram image activities and appearance-related social media consciousness (Choukas-Bradley et al., 2019; Cohen et al., 2017; Di Gesto et al., 2020, 2025), these studies mark a shift from global measures of social media use toward more specific constructs capturing image-based engagement, comparison, and appearance-related self-awareness.

We organized the appearance-focused exposures and activities identified across the included studies into a descriptive taxonomy. This taxonomy was not intended to impose mutually exclusive categories, but rather to clarify the main forms of appearance-focused social media engagement examined in the literature and the psychological mechanisms most commonly associated with them. As shown in Table 2, the included studies could be mapped onto five broad and partially overlapping domains: idealized appearance exposure, fitness-oriented appearance content, appearance-related self-presentation activities, harmful or risk-promoting content, and protective or counter-normative content (Anixiadis et al., 2019; Cohen et al., 2017; Patel et al., 2025; Rodgers et al., 2022; Tiggemann & Zaccardo, 2015).

This taxonomy also clarifies why appearance-focused social media should not be treated as a single homogeneous exposure. Passive viewing of idealized images, active self-presentation through edited photographs, engagement with fitspiration or transformation content, and exposure to body positive or body neutrality material may all fall under the broad umbrella of appearance-focused social media, but they are likely to operate through partly different psychological pathways. Idealized and fitness-oriented content most directly activates comparison and internalization processes (Anixiadis et al., 2019; Fardouly et al., 2018; Tiggemann & Zaccardo, 2015); self-presentation activities are more closely linked to self-objectification, body surveillance, and social-evaluative concerns (Cohen et al., 2017; Fardouly et al., 2015b; Seekis et al., 2020); harmful or risk-promoting content may intensify disordered eating cognitions and negative affect (Jennings et al., 2021; Patel et al., 2025); whereas body positive, body diverse, and body neutrality content may exert protective effects by promoting body appreciation or reducing upward comparison (Rodgers et al., 2022; Rousseau, 2024; Seekis & Lawrence, 2023). At the same time, the boundaries between these categories remain porous, which explains why several studies were coded across more than one content or activity domain.

### 4.3. Mapping of Body Image, Self-Esteem, and Wellbeing Outcomes

The outcomes assessed across the 58 included studies span a wide conceptual territory, and many studies measured several outcomes at once; full study-level mapping is reported in Supplementary Table S3.

Body dissatisfaction is the single most measured construct in this literature. It appears in two forms: as a stable trait and as a state response to acute experimental exposure (Fardouly & Holland, 2018; Tiggemann & Zaccardo, 2015; Tiggemann et al., 2018; see Supplementary Table S3). Closely related outcomes include body satisfaction (Fardouly et al., 2017; Fioravanti et al., 2023; Seekis & Lawrence, 2023; Lee, 2025; Samson & Zaitsoff, 2023), body esteem (Yang et al., 2020; Chang et al., 2019; Rousseau, 2024; Markey & Daniels, 2022), and body appreciation, the positive, acceptance-oriented facet of body image (Rousseau, 2024; Markey & Daniels, 2022; Becker et al., 2025).

A second cluster of outcomes maps onto self-objectification and its behavioral counterpart, body surveillance, examined in 17 studies. These constructs were measured via the Objectified Body Consciousness Scale (OBCS; McKinley & Hyde, 1996); for younger samples, the youth-adapted OBC-Y was the preferred instrument (e.g., Daniels & Robnett, 2021; Slater & Tiggemann, 2015; Choukas-Bradley et al., 2019; see Supplementary Table S3). Body shame, a construct theoretically grounded in objectification theory, surfaces both as an outcome (Galway & Gammage, 2025; Daniels & Robnett, 2021; Slater & Tiggemann, 2015; Markey & Daniels, 2022) and as a mediator in several models, as discussed in Section 4.4. Eating-related outcomes, though not the principal target of this review, recur as downstream consequences: drive for thinness (EDI subscale; e.g., Kim, 2018; Fardouly et al., 2018; Seekis et al., 2021; see Supplementary Table S3), and eating disorder psychopathology more broadly (Fioravanti et al., 2024).

Self-esteem and broader psychological wellbeing are far less consistently assessed. Global self-esteem was used in only 4 studies (Cohen & Blaszczynski, 2015; Kowal et al., 2026; Hai & Yang, 2022; Scully et al., 2023). Depressive symptoms, most often captured through the CES-D, were examined in only a small number of studies (e.g., Etherson et al., 2022; Choukas-Bradley et al., 2019). Anxiety outcomes were somewhat more frequent but still limited: appearance-specific anxiety appeared in several studies (e.g., Zhong, 2022; Yang et al., 2020; Seekis et al., 2020; Patel et al., 2025), whereas generalised anxiety was assessed in only one (GAD-7; Hai & Yang, 2022). Mood outcomes were captured primarily through visual analogue scales or brief PANAS-derived items, almost always in experimental studies tracking acute negative affect after appearance-focused content exposure (e.g., Fardouly & Holland, 2018; Tiggemann & Zaccardo, 2015; Fioravanti et al., 2023; see Supplementary Table S3). A handful of studies examined more specific behavioral outcomes: acceptance of cosmetic surgery (Yue & Tang, 2024) and intentions to alter diet or exercise (Galway & Gammage, 2025; Flynn & Newman, 2023).

The asymmetry is striking. Body image constructs are mapped in depth, often with multiple instruments per study, while self-esteem and general psychological wellbeing, despite occupying a central place in the theoretical framing of much of this research, are measured in only a handful of investigations. This is one of the clearest empirical gaps to emerge from the synthesis.

### 4.4. Mapping of Psychological Mechanisms

Appearance-based social comparison is the most frequently tested mediator across the included literature, and the evidence in its favour is strong. State appearance comparison emerged as a significant mediator in most experimental studies that formally tested mediation models (e.g., Tiggemann & Zaccardo, 2015; Galway & Gammage, 2025; Brown & Tiggemann, 2016; see Supplementary Table S3). In several cases, it fully accounted for the effects of image exposure on body dissatisfaction, negative mood, or self-objectification. Trait appearance comparison tendency, measured through the Physical Appearance Comparison Scale (PACS or PACS-R; Schaefer & Thompson, 2014) or the Upward and Downward Appearance Comparison Scale (UPACS/DACS; O’Brien et al., 2009), more often functioned as a moderator (Fardouly & Holland, 2018; Fardouly et al., 2015a; Seekis et al., 2021; Anixiadis et al., 2019), though a few cross-sectional studies tested it as a mediator (Lee & Lee, 2021; Fardouly & Vartanian, 2015; Fardouly et al., 2015b).

Internalization of culturally promoted appearance ideals, most often assessed via the Sociocultural Attitudes Towards Appearance Questionnaire in its various versions (SATAQ-3, SATAQ-4, SATAQ-4R; Schaefer et al., 2015), is the second pillar of the mediational literature (e.g., Lee & Lee, 2021; Fardouly et al., 2018; see Supplementary Table S3). Internalization rarely operates in isolation: in several studies it works alongside appearance comparison within serial mediation chains, with both processes contributing to body dissatisfaction or drive for thinness (e.g., Fardouly et al., 2018; Yang et al., 2020; Scully et al., 2023).

The third major mediator class is self-objectification and body surveillance, drawing on the OBCS or the SOQ (e.g., Daniels & Robnett, 2021; Slater & Tiggemann, 2015; Choukas-Bradley et al., 2019; Vendemia & Fox, 2024). Some of the most elaborated serial models combine appearance comparison, internalization, body surveillance, and body shame into multi-step pathways linking social media use or photo-based engagement to disordered eating (Slater & Tiggemann, 2015; Yang et al., 2020; Seekis et al., 2020; Lee, 2025; Fioravanti et al., 2024). These integrative models bring Social Comparison Theory and Objectification Theory together within a single explanatory architecture. Body shame itself functions as a downstream mediator in several models, with a particular role in adolescent samples (Daniels & Robnett, 2021; Slater & Tiggemann, 2015).

Beyond these three pathways, a smaller body of work explores less conventional mediators: cognitive emotion regulation strategies such as rumination and catastrophising (McComb & Mills, 2021), psychological investment in appearance (Fioravanti et al., 2024), broad conceptualization of beauty (Rousseau, 2024; Markey & Daniels, 2022), and body acceptance by others (Becker et al., 2025). Yet the centre of gravity is unmistakable: the great majority of mediation analyses converge on the same triad (appearance comparison, internalization, self-objectification) directly mirroring the sociocultural and objectification frameworks discussed in Section 2.

### 4.5. Mapping of Moderating and Protective Factors

Moderation analyses are far less developed than mediation analyses in this literature, and the picture they yield is fragmentary.

Individual-difference variables are the most frequently tested moderators. Trait appearance comparison tendency was examined in several experimental studies with mixed outcomes: it moderated the impact of image exposure on facial dissatisfaction or self-objectification in some (Fardouly et al., 2015a), but failed to reach significance in others (Tiggemann & Zaccardo, 2015; Pryde & Prichard, 2022; Anixiadis et al., 2019). Thin-ideal internalization as a moderator produced equally inconsistent results (Fardouly & Holland, 2018; Cohen & Blaszczynski, 2015; Pryde & Prichard, 2022; Anixiadis et al., 2019). Self-compassion deserves a closer look: in Galway and Gammage (2025), trait self-compassion did not buffer the effects of WIEIAD content on body-related self-conscious emotions, yet in Seekis et al. (2021), specific facets of self-compassion, reduced self-judgment, isolation, and overidentification buffered the indirect effect of fitspiration exposure on body dissatisfaction and drive for thinness. Other moderators surface in single studies only: self-critical perfectionism (Etherson et al., 2022), physical appearance perfectionism (McComb & Mills, 2021), and celebrity worship (Brown & Tiggemann, 2016).

A second strand of evidence shifts the focus from individual traits to the content itself. Body positive content is the most consistently identified protective category: in Fioravanti et al. (2023), daily exposure to body positive Instagram content over 28 days produced the highest growth in positive mood and body satisfaction relative to fitspiration and neutral conditions. In Livingston et al. (2020) and Becker et al. (2025), body positive material was linked to more favourable, though heterogeneous, body image outcomes. Body neutrality content showed a comparable effect in Seekis and Lawrence (2023), eliciting higher functionality appreciation, body satisfaction, and positive mood than thin-ideal content. Self-compassion TikTok content (Seekis & Lawrence, 2023) reduced face-related shame and appearance anxiety relative to beauty content. Family-focused social media engagement (Hogue & Mills, 2019) did not produce the body image decrements observed in the peer-focused condition. These findings remain preliminary and are largely tied to acute experimental exposures, but they point in a consistent direction: the type and framing of appearance-related content, rather than social media exposure, may be the more promising target for protective intervention.

A third strand, much thinner, concerns environmental and behavioral moderators. Account type (following appearance-neutral travel accounts versus health-fitness or celebrity accounts) was tentatively identified as protective in Cohen et al. (2017). Engagement style (active versus passive use, communication-focused versus appearance-focused engagement) was examined longitudinally in Markey and Daniels (2022). Emerging constructs such as social media literacy, broad conceptualization of beauty (Rousseau, 2024; Markey & Daniels, 2022), and body acceptance by others (Becker et al., 2025) appear repeatedly in the discussion sections of the included studies as theoretically relevant protective resources, but they are rarely tested empirically within moderation frameworks.

The mapping reveals a clear gap between theory and empirical investigation. The literature has had much more to say about how appearance-focused social media use harms its users than about under what conditions this harm is attenuated. Closing this gap is arguably one of the most pressing priorities for the field.

**Figure 3:**
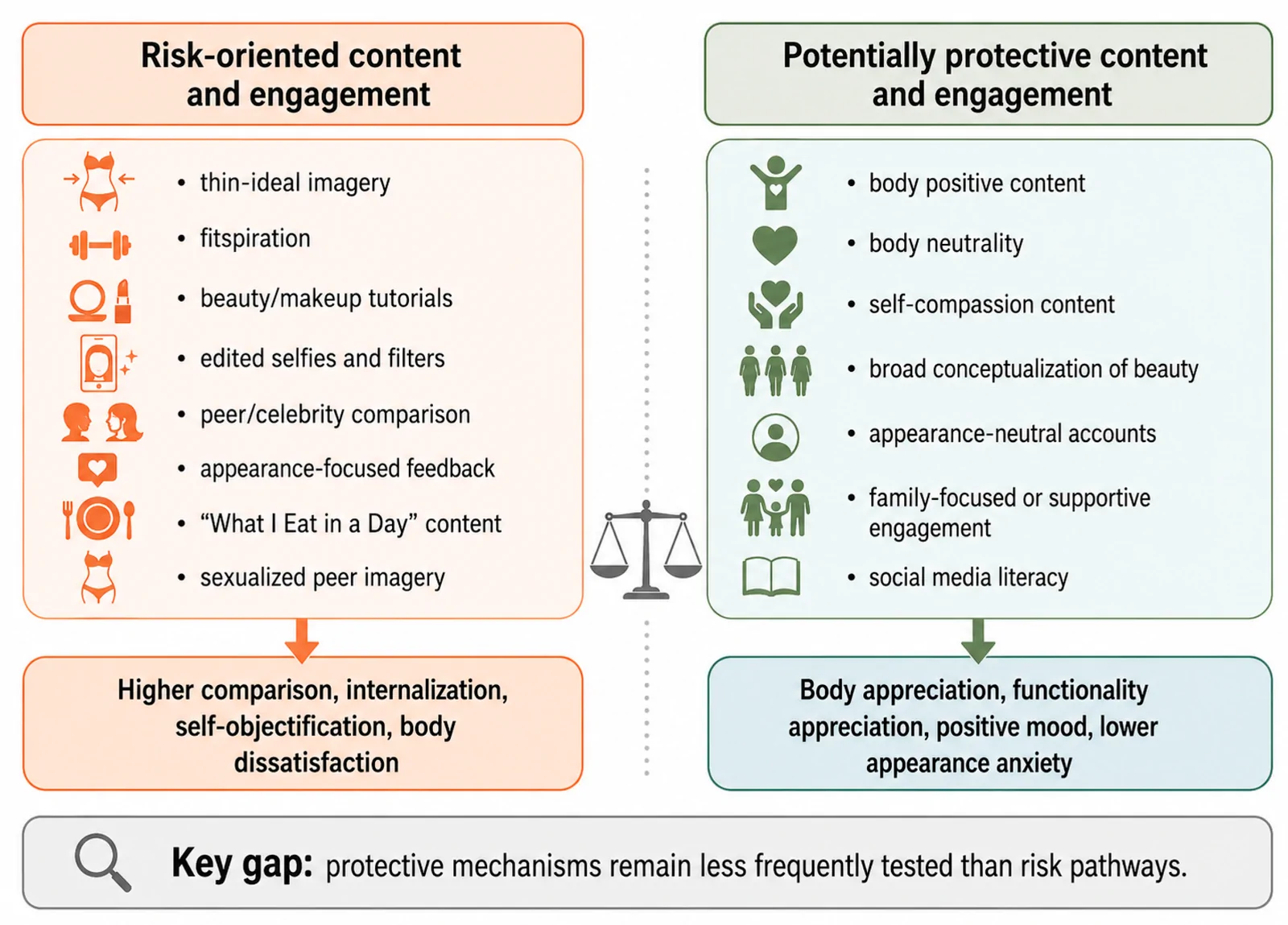
Evidence map of risk-oriented and potentially protective appearance-focused social media content. Note: Risk-oriented content includes thin-ideal, fitspiration, beauty-focused, edited, peer-comparison, and appearance-feedback material, which is generally linked to higher appearance comparison, internalization, self-objectification, and body dissatisfaction. Potentially protective content includes body positive, body neutrality, self-compassion, appearance-neutral, and supportive engagement, which may promote body appreciation, functionality appreciation, positive mood, and lower appearance anxiety. The figure also highlights the relative underdevelopment of research on protective mechanisms.

Because the included studies varied substantially in the types of content and engagement examined, Figure 3 provides an evidence map distinguishing risk-oriented appearance-focused material from potentially protective digital content. This visual synthesis also emphasizes a key asymmetry in the field: harmful pathways have been investigated more extensively than protective or resilience-promoting mechanisms.

### 4.6. Integration of Psychological Mechanisms Across Theoretical Frameworks

Across the included studies, three mechanisms emerged as the most recurrent explanatory pathways: appearance-based social comparison, internalization of appearance ideals, and self-objectification/body surveillance (Fardouly et al., 2018; Scully et al., 2023; Seekis et al., 2020). These mechanisms were rarely isolated from one another. Rather, the mapped evidence suggests a sequential and mutually reinforcing pattern. Exposure to idealized or highly curated images provides comparison targets; repeated upward appearance comparison may increase the internalization of thin, fit, or beauty ideals; and internalized ideals may in turn intensify self-objectification and body surveillance, as girls and young women monitor their bodies from an external evaluative perspective. This proposed pathway is consistent with the convergence of Social Comparison Theory, Objectification Theory, and the Tripartite Influence Model (Festinger, 1954; Fredrickson & Roberts, 1997; Roberts et al., 2022; Thompson et al., 1999).

The strength and direction of these mechanisms varied by content type and engagement style. Idealized celebrity, influencer, and peer images most consistently activated comparison and internalization pathways (Anixiadis et al., 2019; Brown & Tiggemann, 2016; Fardouly et al., 2018). Photo-based self-presentation activities, including selfie editing, posting, and monitoring feedback, were more closely aligned with self-objectification, body surveillance, and contingent self-worth (Cohen et al., 2017; Fardouly et al., 2015b; Seekis et al., 2020). Fitspiration and transformation content appeared to operate through a hybrid mechanism, combining upward comparison with moralized ideals of discipline, health, and bodily control (Samson & Zaitsoff, 2023; Tiggemann & Zaccardo, 2015). Body positive and body neutrality content, by contrast, appeared to attenuate comparison and support body appreciation or self-compassion, although this protective signal remains preliminary (Rodgers et al., 2022; Rousseau, 2024; Seekis & Lawrence, 2023). Overall, the literature supports a multi-mechanism model rather than a single causal pathway.

## 5. Discussion

### 5.1. Summary of Main Findings

This scoping review mapped 58 empirical studies published between 2014 and 2026 on the relationship between appearance-focused social media use, body image, self-esteem, and psychological wellbeing in adolescent girls and young women. Three broad findings stand out.

First, the evidence base is geographically narrow and developmentally skewed. Australia and the United States together account for more than half of the included studies, while sub-Saharan African, Middle Eastern, and Latin American contexts are absent altogether. Within samples, emerging adult women in their twenties are heavily over-represented; younger adolescents (paradoxically the developmental group at greatest risk, according to the very theoretical accounts that motivate this literature (Choukas-Bradley et al., 2022; Smolak, 2004)), appear in only 9 of 58 studies. The mismatch between the developmental framing of the field and the populations studied is one of the most consequential gaps to emerge from the synthesis.

Second, the mediational architecture is remarkably consistent. Across study designs, platforms, and samples, three psychological mechanisms recur as the dominant explanatory pathways: appearance-based social comparison, internalization of culturally promoted appearance ideals, and self-objectification, often combined into serial mediation chains that link social media exposure to body dissatisfaction, drive for thinness, body shame, and disordered eating. The convergence onto this triad, clearly anchored in Social Comparison Theory (Festinger, 1954), Objectification Theory (Fredrickson & Roberts, 1997), and the Tripartite Influence Model (Thompson et al., 1999), gives the field a strong theoretical backbone, but it also raises a question worth posing: are other mechanisms being systematically overlooked because the dominant frameworks define the questions worth asking?

Third, content matters more than time. This is consistent with evidence suggesting that individual socio-emotional abilities and coping strategies are more accurate predictors of problematic internet use than simple connection duration (Tonioni et al., 2018). Studies that focused on total time spent on social networking sites tended to produce weaker and less consistent associations with body-related outcomes, while studies that examined specific content categories (fitspiration, thin-ideal imagery, peer photographs, body positive material, body neutrality, beauty tutorials, WIEIAD videos) found systematically larger and more interpretable effects. Within this content landscape, body positive and body neutrality material emerged as the most promising protective categories, though the evidence rests almost entirely on short-term experimental exposures. By contrast, moderation analyses examining individual differences (self-compassion, perfectionism, comparison tendency) produced mixed results and are heavily underpowered relative to the conceptual ambition of the field.

These three findings reframe what the literature on social media and body image is currently able to tell us. The field has built a strong descriptive and mechanistic account of how appearance-focused content erodes body image in young women in high-income Western contexts. It has been considerably less successful in identifying when, for whom, and under what conditions these effects are amplified or mitigated.

### 5.2. Implications for Research, Clinical Practice, and Prevention

The implications cut across three domains.

For research, the synthesis highlights the value of disaggregating “social media use” into specific content categories and engagement patterns. Studies that adopt this finer-grained approach systematically yield more interpretable findings than those relying on aggregate exposure metrics. The next research generation in this area should treat platform, content type, and engagement style as independent and interacting predictors rather than as proxies for one another. The growing attention to TikTok, body positive content, and culturally distinct platforms such as Xiaohongshu indicates that this shift is already in motion.

For clinical practice, the findings carry several practical implications. First, body image difficulties in adolescent girls and young women cannot be approached without addressing the digital environments in which they unfold. It is therefore crucial for clinicians to recognize internet addiction as a distinct psychopathological condition with specific clinical features that differentiate it from other behavioral addictions (Tonioni et al., 2014). Clinicians working in eating disorder, anxiety, and mood disorder services should routinely assess patterns of appearance-focused social media engagement, with particular attention to the activities (photo editing, comparison with peers and influencers, exposure to fitspiration and idealized imagery) that the literature most consistently links to body dissatisfaction, body surveillance, and disordered eating. Second, the mediational evidence supports targeted clinical work on appearance-based social comparison, internalization of beauty ideals, and self-objectification as transdiagnostic processes relevant across body-image-related presentations. Third, the protective signal associated with body positive and body neutrality content, though preliminary, suggests that clinicians might productively integrate “content reshaping” interventions (curating the feed, unfollowing appearance-focused accounts, intentional exposure to acceptance-oriented material) into broader treatment plans.

For prevention and public health, the implications are even broader. Body image difficulties are a major risk factor for eating disorders, depressive symptoms, and reduced functioning during adolescence and emerging adulthood (Choukas-Bradley et al., 2022; Dane & Bhatia, 2023; Smolak, 2004). Universal prevention programs delivered in schools, covering social media literacy, critical appraisal of digitally edited content, exposure to body-positive and body-diverse material, and skills for managing appearance comparison, have a clear empirical rationale based on the patterns described above. The relative absence of intervention studies from this synthesis (a consequence of the deliberate scoping decision to map correlational and experimental rather than intervention evidence) does not weaken this recommendation; if anything, it underscores how much room remains for designing, testing, and scaling such programs.

### 5.3. Limitations of the Current Evidence Base

Several limitations of the evidence base itself constrain the strength of inferences that can be drawn at present.

The first is methodological. Half of the included studies adopted cross-sectional designs, which by construction cannot disentangle the direction of association between social media use and body image outcomes. The intuitive causal story, appearance-focused exposure leads to body dissatisfaction, is theoretically well-grounded, but reverse pathways (girls already dissatisfied with their bodies may seek out appearance-focused content) and reciprocal dynamics are likely to operate in parallel. Only 4 longitudinal investigations were identified, which is far too few to establish robust temporal patterns. Experimental studies offer cleaner causal inference but typically rely on brief, controlled exposures whose ecological validity is open to question.

A second limitation concerns measurement heterogeneity. The literature relies on a sprawling and often non-interchangeable set of instruments. Body image is operationalised across more than fifteen distinct constructs (body dissatisfaction, body satisfaction, body appreciation, body esteem, body surveillance, body shame, drive for thinness, internalization, self-objectification, functionality appreciation, and others), and the same construct is frequently measured with different tools across studies. This is a major obstacle to cumulative knowledge: when “body image” can mean many different things, meta-analytic integration becomes difficult, and effect-size comparisons across studies risk being misleading.

A third limitation is the under-development of moderation analyses. Despite strong theoretical interest in individual differences, perfectionism, self-compassion, appearance schematicity, social media literacy, and self-esteem, these variables have rarely been tested in adequately powered designs. Several promising moderators (self-compassion, account type, content type) appear in single studies only, leaving the field unable to identify with confidence the conditions under which appearance-focused content is most harmful or most readily resisted.

A fourth limitation concerns the populations sampled. As noted, the literature is skewed toward emerging adult women recruited from undergraduate psychology pools and online platforms, with comparatively little evidence from younger adolescents (the developmental group of greatest theoretical concern), clinical samples, sexual and gender minority populations, or non-Western cultural contexts. The few studies addressing Chinese platforms hint at culturally distinct dynamics (the role of Xiaohongshu in beauty and lifestyle content, for instance) that the broader literature has not yet engaged with.

Finally, a more conceptual limitation deserves mention. The strong convergence of the field around the comparison–internalization–objectification triad, while theoretically coherent, may be narrowing the questions the literature is willing to ask. Alternative mechanisms, affect regulation, identity processes, parasocial relationships with influencers, algorithmic amplification of appearance-focused content, and peer feedback dynamics receive comparatively little empirical attention.

### 5.4. Limitations of the Present Review

Several decisions and constraints in the conduct of this scoping review should be made transparent.

The search was limited to three electronic databases (PubMed, Scopus, Web of Science) and to articles published in English between January 2010 and May 2026. Relevant work indexed in regional databases, published in other languages, or appearing in grey literature was therefore excluded. The exclusion of intervention studies, a deliberate scoping choice aligned with the review’s mapping objective, means that the synthesis does not capture what is known about the modifiability of the observed associations, and conclusions about clinical and preventive practice should be read with this in mind.

The eligibility criterion of female-only samples aged 10–30, while justified by the well-documented gender asymmetry in body image concerns (Fredrickson & Roberts, 1997; Smolak, 2004), necessarily excludes a substantial body of work on adolescent boys, gender-diverse young people, and mixed-gender samples in which female-specific results could in principle be extracted. The decision to require *n* ≥ 50 excluded a small number of qualitative and pilot investigations whose conceptual contributions may nonetheless be relevant.

The methodological profile of the included literature limits the strength of inference. Cross-sectional designs remain predominant and cannot distinguish whether appearance-focused social media engagement contributes to body dissatisfaction, whether girls and young women with pre-existing body image concerns preferentially engage with such content, or whether reciprocal processes unfold over time. Many studies also relied on self-report measures of both exposure and outcome, creating shared-method variance and increasing vulnerability to recall bias and socially desirable responding. Similar methodological limitations have been identified in previous reviews of social media and body image research (Dane & Bhatia, 2023; Vandenbosch et al., 2022). Convenience sampling was common, particularly through undergraduate psychology pools and online recruitment platforms, limiting representativeness. Moreover, the evidence base remains geographically concentrated in Western, educated, industrialized contexts, especially Australia and the United States, with limited representation from non-Western regions. This is especially problematic because body ideals, gender norms, platform cultures, and beauty standards are culturally embedded and may shape both exposure patterns and psychological responses (Choukas-Bradley et al., 2022; Rodgers et al., 2020).

Consistent with PRISMA-ScR and JBI guidance, we did not conduct a formal critical appraisal of the methodological quality of individual studies. This decision reflects the primary aim of a scoping review, which is to map the extent, range, and characteristics of the evidence rather than to determine the certainty of effect estimates or provide pooled causal conclusions. Nevertheless, the absence of formal quality appraisal means that findings from methodologically heterogeneous studies should not be interpreted as carrying equivalent evidential weight. This is in line with scoping review methodology (Peters et al., 2020; Tricco et al., 2018), but it means that the present synthesis treats all included studies as informative regardless of their internal validity. Readers should bear in mind that the picture summarised here reflects the literature as it stands, including its methodological weaknesses, rather than a hierarchy of more or less trustworthy findings. Although two reviewers independently screened all records and extracted all data with substantial inter-rater agreement, some degree of subjective judgement is inherent in the categorisation of content types, mediators, and moderators. The thematic synthesis presented here reflects one defensible organisation of the evidence; alternative thematic groupings might emphasise different patterns.

Meta-analysis was not performed because the included studies were highly heterogeneous in design, exposure definitions, platforms, content categories, outcome constructs, and measurement instruments, consistent with the mapping-oriented purpose of scoping review methodology (Peters et al., 2020; Tricco et al., 2018). Combining such studies quantitatively would risk producing pooled estimates that are statistically possible but conceptually misleading. This limitation is particularly relevant in body image research, where constructs such as body dissatisfaction, body satisfaction, body appreciation, body surveillance, body shame, self-objectification, drive for thinness, appearance comparison, and internalization of appearance ideals are related but not interchangeable.

As already outlined, the evidence base remains dominated by cross-sectional designs and self-report measures. Cross-sectional studies cannot establish temporal ordering and are vulnerable to reverse or reciprocal associations: girls and young women with pre-existing body dissatisfaction may be more likely to seek, attend to, or be algorithmically exposed to appearance-focused content. Self-report measures are also vulnerable to recall bias, social desirability, and differences in how participants interpret platform use, appearance comparison, and body image constructs (Dane & Bhatia, 2023; Vandenbosch et al., 2022). Future studies should prioritize longitudinal, ecological momentary assessment, experimental, and mixed-method designs, together with more consistent and developmentally validated measurement frameworks. A further limitation concerns the developmental distribution of the available evidence. Although the review was designed to cover adolescent girls and young women, the included studies were heavily weighted toward emerging adulthood. Only a small minority of studies focused specifically on girls aged 10–17 years. This is a critical gap because the developmental processes most often invoked to explain vulnerability to appearance-focused social media (pubertal change, identity formation, heightened peer sensitivity, and increasing appearance-based self-evaluation) are especially salient during early and middle adolescence (Choukas-Bradley et al., 2022; Smolak, 2004). Future studies should therefore recruit age-stratified samples and report findings separately for early adolescence, late adolescence, and emerging adulthood, rather than treating these periods as interchangeable.

### 5.5. Future Directions

The synthesis suggests several priorities for the next phase of research.

A priority is methodological diversification. The field needs more longitudinal investigations, particularly multi-wave studies and intensive longitudinal designs (ecological momentary assessment, experience sampling) capable of tracking within-person dynamics over meaningful timescales. The four longitudinal studies identified here (Etherson et al., 2022; Fardouly et al., 2017; Fioravanti et al., 2023; Markey & Daniels, 2022) provide an instructive template but are too few to support robust generalisations. Pre-registered experiments with greater ecological validity (for example, manipulating the participant’s own feed over days or weeks rather than presenting brief controlled stimuli) would also represent a substantial step forward.

A second priority is demographic and cultural broadening. Research on younger adolescent girls (10–14), clinical and at-risk samples, and non-Western contexts is urgently needed. The current near-absence of work from sub-Saharan Africa, the Middle East, and Latin America is a major gap, particularly given the global reach of platforms such as Instagram and TikTok. The small but distinct evidence base on Chinese platforms (Song et al., 2025; Yue & Tang, 2024; Zhong, 2022; Zhou et al., 2024) suggests that culturally specific platforms produce culturally specific dynamics worth examining.

A third priority is conceptual integration. The proliferation of body image constructs and measurement tools is a known problem in the field but remains unaddressed. A coordinated effort (perhaps through Delphi-style consensus initiatives) to converge on a core set of constructs and validated instruments would substantially enhance the cumulative power of future research. Beyond convergence, work that explicitly tests competing or complementary mechanisms (e.g., comparison vs. parasocial vs. affect-regulation pathways) within the same studies would help adjudicate among theoretical frameworks that currently coexist with little empirical arbitration.

A fourth priority is the empirical investigation of protective factors. The signals around body positive content, body neutrality content, self-compassion, social media literacy, and the broad conceptualization of beauty are theoretically compelling but rest on a thin evidence base. Adequately powered experimental and longitudinal studies testing these constructs as moderators, and intervention studies translating them into prevention and treatment protocols, should be a central focus of the next research generation.

A fifth priority is engagement with the algorithmic dimension of contemporary social media. The included studies treat platforms primarily as exposure environments; few engage with the active, personalised role that recommendation algorithms play in determining which appearance-focused content reaches which users. As TikTok and Instagram increasingly shape feeds through algorithmic curation, this dimension is no longer optional. Research designs that incorporate algorithmic dynamics, for example, by manipulating recommendation streams or examining trajectories of exposure over time, represent an important methodological and conceptual frontier for the field. The rapidly changing visual ecology of social media requires specific attention. Contemporary platforms increasingly combine image-based content, short-form video, appearance filters, and personalized recommendation processes, potentially altering both the intensity and type of appearance-related exposure. These platform characteristics may reinforce appearance comparison, internalization, and self-objectification pathways, but they remain insufficiently captured by research based on global measures of social media use or static image exposure (Choukas-Bradley et al., 2022; Vandenbosch et al., 2022). Future research should therefore examine algorithmically curated exposure, digitally modified visual content, and short-form video environments as distinct components of the contemporary appearance-focused social media landscape.

## 6. Conclusions

This scoping review synthesized 58 empirical studies published between 2014 and 2026 on the relationship between appearance-focused social media use, body image, self-esteem, and psychological wellbeing in adolescent girls and young women. Three patterns define the current state of the field.

The first is convergence. Across heterogeneous designs, platforms, and samples, the literature consistently identifies appearance-based social comparison, internalization of culturally promoted appearance ideals, and self-objectification as the dominant psychological mechanisms through which exposure to appearance-focused content erodes body image. This convergence offers a coherent theoretical scaffold for clinical and preventive work targeting body image difficulties in young women.

The second is imbalance. The evidence base concentrates heavily on emerging adult women in Australia and the United States, leaving younger adolescent girls (the developmental group at greatest theoretical risk) and non-Western cultural contexts substantially under-represented. Methodologically, the field continues to rely on cross-sectional designs and short experimental exposures, with longitudinal investigations of within-person dynamics remaining scarce. Measurement heterogeneity across more than fifteen distinct body image constructs further limits the cumulative power of the evidence.

The third is opportunity. The emerging signals on body positive and body neutrality content, on protective individual differences such as self-compassion and broad conceptualization of beauty, and on the modifiability of online environments through curating engagement and content type, point toward a generation of research and practice that moves beyond documenting harm. The challenge for the next phase of work is to convert these signals into adequately powered, developmentally and culturally diversified studies, and into prevention and clinical protocols capable of reaching the young women whose body image and wellbeing are most at stake in contemporary appearance-focused digital environments.

## Figures and Tables

**Table 1:** Summary characteristics of the 58 included studies.

Characteristic	n (%)
**Geographical region**	
Australia	17 (29.3)
United States	13 (22.4)
China	5 (8.6)
Canada	4 (6.9)
United Kingdom	4 (6.9)
South Korea	3 (5.2)
Italy	2 (3.4)
Singapore	2 (3.4)
Other (Poland, Croatia, Ireland, Belgium)	4 (6.9)
Multinational	4 (6.9)
**Study design**	
Cross-sectional	29 (50.0)
Experimental	25 (43.1)
Longitudinal/intensive longitudinal	4 (6.9)
**Developmental stage of sample**	
Younger adolescents (10–17 years)	9 (15.5)
Emerging adults/young women (18–30+ years)	49 (84.5)
**Sample size**	
<200	24 (41.4)
200–499	30 (51.7)
≥500	4 (6.9)
**Social media platform examined**	
Instagram	34 (58.6)
Facebook	18 (31.0)
TikTok	8 (13.8)
Snapchat	5 (8.6)
Chinese platforms (Weibo, WeChat, Xiaohongshu)	4 (6.9)
YouTube	3 (5.2)
**Outcome domains assessed**	
Body dissatisfaction	26 (44.8)
Positive body image (satisfaction, appreciation, esteem)	18 (31.0)
Self-objectification/body surveillance	17 (29.3)
Body shame	5 (8.6)
Drive for thinness/disordered eating	14 (24.1)
Mood/affect	15 (25.9)
Anxiety	5 (8.6)
Self-esteem (global)	3 (5.2)
Depressive symptoms	3 (5.2)

Note: Percentages are calculated over the total of 58 included studies. a Categories are not mutually exclusive: studies examining more than one platform, or assessing more than one outcome domain, are counted under each applicable category. Full study-level details are reported in Supplementary Tables S2 and S3.

**Table 2:** Conceptual taxonomy of appearance-focused social media engagement across included studies.

Category	Examples from Included Studies	Main Hypothesized Mechanisms	Typical Outcomes Examined
Idealized appearance exposure	Thin-ideal images, beauty-focused images, celebrity images, influencer images, curated peer images	Upward appearance comparison; internalization of appearance ideals	Body dissatisfaction; lower body satisfaction; negative affect; reduced self-esteem
Fitness-oriented appearance content	Fitspiration, exercise-related images, weight-loss transformations, “What I eat in a day” videos	Appearance comparison; internalization of fit/thin ideals; moralized body discipline; body monitoring	Body dissatisfaction; drive for thinness; disordered eating attitudes; body surveillance
Appearance-related self-presentation activities	Selfie taking, photo editing, posting, tagging, monitoring likes and comments, appearance-related feedback-seeking	Self-objectification; body surveillance; contingent self-worth; social-evaluative concern	Body surveillance; body shame; self-objectification; body dissatisfaction
Harmful or risk-promoting content	Thinspiration, pro-anorexia content, restrictive eating imagery, extreme body ideals	Thin-ideal internalization; negative affect; anxiety; disordered eating cognitions	Drive for thinness; eating-disorder symptoms; anxiety; negative mood
Protective or counter-normative content	Body positivity, body neutrality, body diversity, self-compassion content	Body appreciation; self-compassion; reduced upward comparison; resistance to ideal internalization	Body appreciation; body satisfaction; positive affect; reduced body dissatisfaction

Note: Categories are descriptive and partially overlapping rather than mutually exclusive. The categories and associated mechanisms and outcomes were synthesized from representative studies of idealized appearance exposure (Anixiadis et al., 2019; Brown & Tiggemann, 2016), fitness-oriented content (Pryde & Prichard, 2022; Samson & Zaitsoff, 2023; Tiggemann & Zaccardo, 2015), appearance-related self-presentation (Cohen et al., 2017; Lee & Lee, 2021), harmful or risk-promoting content (Jennings et al., 2021; Patel et al., 2025), and protective or counter-normative content (Rodgers et al., 2022; Rousseau, 2024; Seekis & Lawrence, 2023). Fitspiration may combine health-oriented messaging with thin-ideal or muscular-ideal imagery; body positive content may still reproduce conventional attractiveness norms; and influencer or peer images may function simultaneously as idealized exposure and comparison targets.

## Data Availability

All data analysed in this scoping review derive from previously published studies cited in the references. The review protocol was registered on the Open Science Framework (OSF; https://doi.org/10.17605/OSF.IO/BJZGX). The data charting form and extracted data are available in the Supplementary Materials.

## Supplementary Materials

The following supporting information can be downloaded at https://www.mdpi.com/article/10.3390/ejihpe16080108/s1. Table S1: Full electronic search strategy; Table S2: Characteristics of included studies; Table S3: Outcomes, instruments, mechanisms, and theoretical frameworks of included studies; Table S4: PRISMA-ScR Checklist.
