# Supplementary material for: Bodies on Display: A Scoping Review of Appearance-Focused Social Media, Body Image, Self-Esteem, and Psychological Wellbeing in Adolescent Girls and Young Women
Source: Eur J Investig Health Psychol Educ. 2026 Jul 23;16(8):108. doi: 10.3390/ejihpe16080108 (PMC13512515; doi:10.3390/ejihpe16080108)
Supplement: Supplementary file 1 [file ejihpe-16-00108-s001.zip › Table_S1_Search_Strategy.pdf]

Table S1. Full electronic search strategy

Bodies on Display: A Scoping Review of Appearance-Focused Social Media, Body Image, Self-Esteem, and Psychological Wellbeing in Adolescent Girls and Young Women

The search strategy combined four conceptual blocks with the Boolean operator AND: (i) social media platforms and activities, (ii) body image, self-objectification, and self-esteem constructs, (iii) the target population, and (iv) appearance-focused framing terms. The syntax was adapted to the requirements of each database. The full search strings, applied limits, and number of records retrieved are reported below.

1. PubMed

Date of search: between April 1 and May 15, 2026

Search string:

```
("social media"[MeSH Terms] OR "social media"[tiab] OR "social networking"[tiab]
OR Instagram[tiab] OR TikTok[tiab] OR "image-based platform*"[tiab])
AND
("body image"[MeSH Terms] OR "body image"[tiab] OR "body dissatisfaction"[tiab]
OR "self-objectification"[tiab] OR "body surveillance"[tiab]
OR "self esteem"[MeSH Terms] OR "self-esteem"[tiab])
AND
("adolescent"[MeSH Terms] OR adolescen*[tiab] OR "young women"[tiab]
OR "young adult women"[tiab] OR girl*[tiab])
AND
("2010"[Date - Publication] : "2026"[Date - Publication])
AND
("appearance-focused" OR "appearance-related" OR "appearance comparison")
```

Limits: Publication date 2010–2026; MeSH and title/abstract [tiab] fields.

Records retrieved: 108

2. Scopus

Date of search: April–May 2026

Search string:

```
TITLE-ABS-KEY ( ( "social media" OR "social networking" OR Instagram OR TikTok
OR "image-based platform*" )
AND ( "body image" OR "body dissatisfaction" OR "self-objectification"
OR "body surveillance" OR "self-esteem" )
AND ( adolescen* OR "young women" OR girl* )
AND ( "appearance-focused" OR "appearance-related" OR "appearance comparison" ) )
AND PUBYEAR > 2009 AND PUBYEAR < 2027
AND ( LIMIT-TO ( LANGUAGE,"English" ) )
AND ( LIMIT-TO ( DOCTYPE,"ar" ) OR LIMIT-TO ( DOCTYPE,"re" ) )
```

Limits: Publication years 2010–2026; English language; document types: articles (ar) and reviews (re); fields: title, abstract, keywords.

Records retrieved: 196

3. Web of Science (Core Collection)

Date of search: April–May 2026

Search string:

```
TS=( ("social media" OR "social networking" OR Instagram OR TikTok
OR "image-based platform*")
AND ("body image" OR "body dissatisfaction" OR "self-objectification"
OR "body surveillance" OR "self-esteem")
AND (adolescen* OR "young women" OR girl*)
AND ("appearance-focused" OR "appearance-related" OR "appearance comparison") )
```

Limits: Topic (TS) field = title, abstract, author keywords, Keywords Plus. Timespan 2010–2026, English language, and document types (articles/reviews) applied via the Web of Science interface [to be confirmed by the author].

Records retrieved: 173

Summary of records

Source	Records
PubMed	108
Scopus	196
Web of Science	173
**Total records identified**	**477**
Duplicates removed	227
Records after deduplication	250
**Studies included in the review**	**58**

Note. The same four-block structure was applied across all three databases, with syntax adapted to each platform. Asterisks (*) denote truncation. The exact date of the most recent search should be specified before submission (PRISMA-ScR item 7). For Web of Science, any timespan, language, or document-type limits applied through the interface should be confirmed and reported here to ensure full reproducibility.
