# Supplementary material for: Bodies on Display: A Scoping Review of Appearance-Focused Social Media, Body Image, Self-Esteem, and Psychological Wellbeing in Adolescent Girls and Young Women
Source: Eur J Investig Health Psychol Educ. 2026 Jul 23;16(8):108. doi: 10.3390/ejihpe16080108 (PMC13512515; doi:10.3390/ejihpe16080108)
Supplement: Supplementary file 1 [file ejihpe-16-00108-s001.zip › Table_S2_Study_Characteristics.pdf]

## Table S2. Characteristics of the 58 included studies

*Bodies on Display: A Scoping Review of Appearance-Focused Social Media, Body Image, Self-Esteem, and Psychological Wellbeing in Adolescent Girls and Young Women*

| ID | Author (Year) | Country | Study design | Recruitment | N | %F | Mean age | Platform(s) | Content/activity |
|---|---|---|---|---|---|---|---|---|---|
| S001 | Lee & Lee (2021) | South Korea | Cross-sectional, online survey | Online (survey company) | 385 | 100% | M=29.83 (SD=4.93); range 20–39 | Instagram (85.7%), YouTube (71.4%), Facebook (59.5%), blogs (62.3%) | Appearance-related photo activity (posting, viewing, commenting on photos); photo-editing behavior |
| S002 | Fardouly & Holland (2018) | USA | Experimental, between-subjects, online | Online (Amazon Mechanical Turk) | 164 | 100% | M=23.09 (SD=1.69); range 18–25 | Instagram | Idealized Instagram images of attractive women (with/without self-disclaimer comments) vs travel control images |
| S003 | Roorda et al. (2026) | Canada | Experimental, between-subjects, online (2×2 ANOVA) | University (introductory psychology classes, Toronto Metropolitan University) | 256 | 100% | M=19.80 (SD=3.08); range 17–30 | TikTok | WIEIAD videos (with/without thin-ideal body depiction) vs interior design control videos (with/without thin-ideal body depiction) |
| S004 | Yue & Tang (2024) | China | Cross-sectional, online survey (SEM, multi-group analysis) | Online (Sojump platform, snowball sampling via WeChat) | 554 | 100% | M=21.6 (SD=2.1); range 18–29 | Chinese social media (WeChat, Douban; general image-sharing platforms) | Idealized appearance-related content (slim bodies, flawless skin, etc.) vs non-idealized content (diverse bodies, imperfections) |
| S005 | Cohen & Blaszczynski (2015) | Australia | Experimental, between-subjects, online (2×2 mixed design) | University (first year psychology undergraduates, University of Sydney) | 193 | 100% | M=19.32 (SD=3.47); range 17–46 | Facebook (vs conventional media) | Thin-ideal Facebook mock profile images with peer comments vs conventional media thin-ideal images (models/celebrities) |
| S006 | Fardouly et al. (2017) | Australia | EMA (Ecological Momentary Assessment), 5 days, 5 signals/day | University (first year psychology students, large public university) | 146 | 100% | M=19.24 (SD=2.24) | Social media (e.g., Facebook); traditional media (TV/movies, magazines, billboards); in person | Naturalistic appearance comparisons (upward, lateral, downward) in different contexts in everyday life |
| S007 | Zhong (2022) | China | Cross-sectional, online survey (regression analysis) | Online (www.wjx.cn, Chinese survey platform) | 367 (valid responses) | 100% | Not specified | Xiaohongshu (Chinese UGC social media platform) | Use frequency/duration of Xiaohongshu; exposure to "ideal beauty" content (makeup, fashion, skincare, body ideals promoted by internet celebrities) |
| S008 | Kim (2018) | South Korea | Cross-sectional, online survey (mediation analysis) | Online (Korean research company registered panels) | 305 | 100% | M=23.44 (SD=2.64); range 18–29 | Facebook | Profile maintenance (posting photos, editing profile) and social grooming (liking, commenting on friends' profiles) |
| S009 | Kowal (2026) | Poland | Pre-registered experiment (between-subjects: social media models vs. abstract paintings) | University hallways (University of Wroclaw, Poland) | 164 | 100% | M=22.09 (SD=4.59) | Instagram (idealized social media models) | Viewing idealized social media model photos (10 images with simulated likes/comments interface) vs. neutral abstract paintings; ratings of visual appeal, like/share likelihood |
| S010 | Etherson (2022) | UK | Multi-wave longitudinal study (4 weekly timepoints; multilevel modeling) | High school (classroom-based) | 135 | 100% | M=14.70 (SD=0.46) | Social media (>80% of appearance comparisons occurred on social media) | Appearance-focused upward social comparison (comparing own appearance with others perceived as more attractive on social media) |

| ID | Author (Year) | Country | Study design | Recruitment | N | %F | Mean age | Platform(s) | Content/activity |
|---|---|---|---|---|---|---|---|---|---|
| S011 | Tiggemann (2015) | Australia | Between-subjects experiment (fitspiration images vs. travel images control) | University laboratory (Flinders University, South Australia) | 130 | 100% | M=19.91 (SD=2.80) | Instagram | Viewing 18 Instagram fitspiration images (thin/toned women exercising or in fitness clothing, with inspirational quotes) vs. 18 travel images (control) |
| S012 | Seekis (2023) | Australia | Between-subjects experiment (3 conditions: beauty TikToks vs. self-compassion TikToks vs. travel control TikToks) | University laboratory (Griffith University, Australia) | 115 | 100% | M=19.37 (SD=1.89) | TikTok | Viewing 7-min beauty TikTok compilation (makeup tutorials, skincare routines, cosmetic procedure before/after) vs. 7-min self-compassion TikToks vs. 7-min travel control TikToks |
| S013 | Galway (2025) | Canada / USA | Between-subjects experiment (3 conditions: WIEIAD TikTok videos with calories vs. without calories vs. travel control) | Online (Cloud Research Connect / Qualtrics) | 335 | 100% | M=25 (SD=3.24); range 18-30 | TikTok | Viewing 7-min #WhatIEatInADay TikTok videos (with or without calorie labels added) showing women preparing/eating food in an aesthetic manner, including appearance-focused/objectifying content; vs. 7-min travel control videos |
| S014 | Fioravanti (2023) | Italy | Quasi-experimental intensive longitudinal study (ESM, 28 consecutive days; 3 conditions: body-positive vs. fitspiration vs. neutral Instagram content; multilevel regression / linear growth model) | Instagram (recruited via Instagram announcements) | 122 | 100% | M=22.29 (SD=2.26) | Instagram | Daily exposure to researcher-created Instagram profiles: body-positive (#BoPo hashtags + 1 post + 3 stories/day) or fitspiration (#fitspo) or neutral (animals, nature, travel) content for 28 days |
| S015 | Walker (2015) | USA | Cross-sectional online survey (hierarchical regression + post-hoc mediation; PROCESS macro) | University (online survey, large southeastern university) | 128 | 100% | Not reported (range 18-23) | Facebook | Facebook intensity (emotional connection + daily integration); online physical appearance comparison; online fat talk on Facebook |
| S016 | Belmonte (2024) | USA | Cross-sectional survey (3-stage serial mediation model; path analysis via AMOS; bootstrapped serial mediation) | University (large Midwestern university; online survey) | 189 | 100% | M=20.78 (SD=2.00); range 18-30 | Instagram | Instagram use (time per day) and intensity; viewing celebrity/influencer content promoting voluptuous/curvy body ideal (e.g., Kardashian-style imagery) |
| S017 | Dajches (2025) | USA | Cross-sectional survey (path analysis with bootstrapped mediation; AMOS) | Mixed recruitment: university participant pool + Reddit + Prolific (online survey) | 424 | 100% | M=22.53 (SD=3.31); range 18-32 | TikTok | Leaving appearance-related comments on others' TikTok videos; following celebrity TikTok accounts (models, singers, actors, reality TV); following health/fitness TikTok accounts |
| S018 | Chansiri et al. (2020) | USA | Experiment (post-test only, between-subjects, 3 conditions: fitspiration vs. thinspiration vs. control scenery images) | Large public university; convenience sample; extra course credits as incentive | 221 | 100% | M=21 (SD=1.75) | Instagram | Viewing 10 fitspiration images (thin & toned women exercising, active outfits, visible muscles) or 10 thinspiration images (extra-thin women, no exercise, non-active attire) or 10 control scenery images. Sourced via #fitspiration/#fitspo and |

| ID | Author (Year) | Country | Study design | Recruitment | N | %F | Mean age | Platform(s) | Content/activity |
|---|---|---|---|---|---|---|---|---|---|
| | | | | | | | | | #thinspiration/#thinspo; presented in fake Instagram frames. |
| S019 | Flynn & Newman (2023) | UK (Scotland) | Experiment (between-subjects, 3 image conditions; mixed ANOVA pre/post design) | Online recruitment via social media (Instagram, Facebook, Twitter, LinkedIn, Reddit) | 404 | 100% | M=25.79 (SD=3.45); range 18–30 | Instagram | Before and after weight loss transformation images (BAWLI): 16 side-by-side images sourced via #weightlosstransformation, #beforeandafterweightloss, #beforeandafterweightlosstransformation (women in workout clothes or underwear/bikinis; faces blurred; diverse ethnicity, body sizes). Condition 2: same images cropped and presented individually in random order (no transformation context). Control: 16 travel images from #womenwhoexplore, #sheexplores, #sheisnotlost. |
| S020 | Daniels & Robnett (2021) | USA | Cross-sectional survey (path analysis; Mplus) | Online panel (Qualtrics); parents of middle school girls contacted; small monetary incentive | 243 (of initial pool 477) | 100% | Range 11-14 (mean NR) | Facebook (primarily; also general social media) | Appearance-focused social media behaviors: posting/updating profile photo, tagging self in photos, and other photo-specific activities on Facebook (photo subscale of the FBQ; Meier & Gray, 2014). No experimental image exposure — survey-based measurement of habitual behavior. |
| S021 | Fardouly et al. (2018) | USA & Australia | Cross-sectional survey (correlational; multiple mediation via PROCESS) | USA: Amazon Mechanical Turk (MTurk); Australia: female undergraduates at a large university | 276 | 100% | M=22.83 (SD=3.57); range 18–25 | Instagram | Overall Instagram use (frequency of checking + daily time); viewing fitspiration images on Instagram (photos of fit people, gym, healthy food, inspirational fitness/diet quotes); appearance comparisons to specific target groups on Instagram (family, friends, acquaintances, strangers, celebrities, self, women in fitspiration images) |
| S022 | Fardouly et al. (2015) | UK | Experiment (between-subjects, 3 conditions; 2-part design; moderated regression) | University (UK): psychology student pool (course credit) and flyer recruitment (paid £10) | 112 | 100% | M=20.46 (SD=1.71); range 17–25 | Facebook; online fashion magazine (Cosmopolitan UK); appearance-neutral control website (home craft) | 10-minute free browsing of own Facebook account (peers' images/profiles, status updates) vs. UK Cosmopolitan magazine website (models/celebrities) vs. appearance-neutral control website (home craft). Naturalistic browsing — no specific content prescribed. |
| S023 | Slater & Tiggemann (2015) | Australia | Cross-sectional survey (path analysis; AMOS) | 18 secondary schools across South Australia (metropolitan + rural; private + public); parental consent obtained | 1087 | 100% | M=13.68 (SD=.70); range 12–16 | Social networking sites (Facebook, MySpace); magazines; television; general Internet | Self-reported exposure to: (1) appearance-focused TV programs (10 programs, OzTam ratings); (2) appearance-focused magazines (11 magazines; e.g., Dolly, Cosmopolitan, Vogue); (3) general Internet use (hrs/day); (4) SNS use (Facebook + MySpace, mins/day). Also: extracurricular activities (embodying, appearance-focused, other) and positive/negative appearance-related comments (from girls, boys, parents). |
| S024 | Zhou et al. (2024) | China | Cross-sectional survey | Three universities and one professional college in China | 2126 | 100% | M=23.36 | Sina Weibo, WeChat, TikTok, | Beauty-related content (beauty bloggers, models, celebrities) |

| ID | Author (Year) | Country | Study design | Recruitment | N | %F | Mean age | Platform(s) | Content/activity |
|---|---|---|---|---|---|---|---|---|---|
| | | | | | | | | LittleRedBook (Xiaohongshu) | |
| S025 | Zulec Ivankovic et al. (2024) | Croatia | Cross-sectional survey (path analysis) | Catholic University of Croatia; snowball sampling via psychology students sharing the survey link | 308 | 100% | M=21.27 (SD=1.34) | Instagram (primary focus); general social media/smartphone use | Appearance-focused content; beauty ideals (thinness/low-fat; muscularity/athletic) |
| S026 | Ferdousi et al. (2023) | Australia | Cross-sectional survey; serial mediation (SEM/AMOS) | Undergraduate psychology course participant pool at Griffith University (Queensland) | 284 | 100% | M=18.29 (SD=0.60); range 17-19 | Instagram (advertising content specifically) | Thin models (slim body type) and curvy models (large breasts/buttocks, wide hips); Instagram advertisements |
| S027 | Livingston et al. (2020) | Australia | Experimental (laboratory-based; 3-condition between-subjects) | University laboratory; first-year undergraduate psychology students, University of Queensland | 201 | 100% | M=18.93 (SD=1.24); range 18-25 | Instagram (simulated; images embedded in Instagram border) | Idealized/edited images of a thin, attractive Caucasian woman (appearance-focused); body-positive images; appearance-neutral images (sunsets/landscapes) |
| S028 | Hogue & Mills (2019) | Canada | Experimental (2-condition between-subjects; 2x2 mixed ANOVA) | York University undergraduate psychology participant pool (Toronto); online experiment | 118 (final; 125 recruited) | 100% | M=19.59 (SD=2.15); range 18-25 | Facebook and Instagram (participants used own real accounts) | Appearance-focused content on peers' profiles (photos); family member social media content (control condition) |
| S029 | Hai & Yang (2022) | China | Cross-sectional survey; chain mediation analysis (PROCESS macro, Hayes) | Online survey platform (wjx.cn); recruited female college students or graduates via social media ads | 320 | 100% | M=21.6 (SD=3.2); range 17-39 | Social network sites (general; not platform-specific) | Appearance-focused content (selfies, videos); body image comparison content on SNS |
| S030 | Choukas-Bradley et al. (2019) | USA | Cross-sectional survey; 2 path analyses (SEM; Mplus 7.4) | Large university in the southeastern United States; undergraduate psychology courses (online survey) | 339 | 100% | M=18.35; range 18-19 | Facebook, Instagram, Snapchat, Twitter, and other photo-based social media (general) | Photo-based appearance-focused content; appearance ideals visible to social media audience |
| S031 | Fardouly & Vartanian (2015) | Australia | Cross-sectional correlational survey; mediation analysis (bootstrapping; Preacher & Hayes) | Large public university in eastern Australia; online psychology participant pool (course credit) | 227 | 100% | M=19.13 (SD=2.21) | Facebook | Photos of peers (close friends, distant peers), family members, and celebrities on Facebook; Facebook appearance comparisons |
| S032 | Seekis & Lawrence (2023) | Australia | Experimental (3-condition between-subjects; ANCOVAs/ANOVAs) | Large public university in Australia; introductory psychology courses (face-to-face recruitment) | 189 | 100% | M=19.25 (SD=1.98); range 17-28 | TikTok (12-min compilation videos uploaded to YouTube for experiment) | Body neutrality content (diverse body types; non-judgmental functionality focus); thin ideal content (slim models); appearance-neutral content (nature/animals/food; control) |

| ID | Author (Year) | Country | Study design | Recruitment | N | %F | Mean age | Platform(s) | Content/activity |
|---|---|---|---|---|---|---|---|---|---|
| S033 | Yang et al. (2020) | Singapore | Cross-sectional survey | Local community (female adolescents recruited in exchange for monetary reward) | 100 | 100% | M=15.07 (SD=1.33); range 13-18 | Facebook, Instagram, Snapchat, Twitter; smartphones (general screen time + specific activities) | Appearance-related media content (thin models, idealized images, celebrity photos); visually enhanced user-generated content. Passive consumption: social media use, website browsing, watching TV shows, listening to music, texting, emailing, photography, online shopping |
| S034 | Seekis et al. (2020) | Australia | Cross-sectional survey; structural equation modeling (SEM; AMOS 25) | University (Griffith University, Gold Coast); first-year psychology course (course credit); online questionnaire | 338 | 100% | M=19.12 (SD=2.10); range 17-25 | Instagram (44%), Snapchat (29%), Facebook (24%), other 3% (Pinterest, Tumblr); general SNS use | Celebrity/fashion/beauty (CFB) content on SNSs (idealized images of celebrities, fashion influencers, beauty/make-up); fitspiration-related content (fitness, sports stars, weight loss); importance of likes and comments (ILC) received on own photos. Passive browsing/following of appearance-related accounts |
| S035 | Fardouly et al. (2015) | UK | Cross-sectional survey; mediation analysis (bootstrapping; Preacher & Hayes, 2008; 5,000 resamples) | University (UK): psychology student pool (course credit, n=53) and flyer recruitment around university (paid 10 GBP, n=97) | 150 | 100% | M=20.52 (SD=1.73); range 17-25 | Facebook; Internet (general); television; music videos; print fashion magazines | Sexually objectifying/appearance-focused media content on Facebook (images of peers, family, celebrities, self) and in fashion magazines; general appearance-focused content across media types. Passive use: time spent on Facebook per day + frequency of checking; magazine reading frequency; TV/Internet/music video time |
| S036 | Song et al. (2025) | China | Experiment (between-subjects; 5 conditions; one-way ANOVA + LSD post-hoc; online via Wenjuanxing platform) | Three universities in mainland China (public university, private university, vocational college); recruited via university networks and student platforms | 369 | 100% | M=20.01 (SD=2.03); range 18-26 | Xiaohongshu (Little Red Book; Chinese social media platform); simulated social media images | 5 image conditions (5 images per condition, 30s minimum exposure each): (1) thin-ideal posed (static stylized body display); (2) thin-ideal functional (thin models exercising/active); (3) full-figured posed (static display, BMI>25 models); (4) full-figured functional (full-figured models active); (5) neutral scenery (nature/urban landscapes; control). All images sourced from Xiaohongshu; faces obscured; standardized attire |
| S037 | Seekis et al. (2021) | Australia | Cross-sectional survey; mediation + moderated mediation (PROCESS macro v3.4 models 4 and 8; 5,000 bootstrap samples) | University (Griffith University, Gold Coast); first-year psychology course (course credit); online questionnaire | 338 | 100% | M=19.12 (SD=2.10); range 17-25 | Instagram and other SNSs (general fitspiration content) | Fitspiration content: images of idealized thin/athletic/toned female body types, fitness routines, diets, clean eating, weight loss content, female sports athletes, fitspiration personalities. Passive browsing/following of fitspiration-related accounts on SNSs (5-item scale, 1=never to 5=daily) |
| S038 | Lee (2022/2025) | South Korea | Cross-sectional survey; structural equation modeling (SEM; AMOS 25; bootstrapping for indirect effects, 10,000 samples) | Online research firm panel (Korea); Korean women in their 20s who use Instagram daily; self-administered online questionnaire | 321 | 100% | M=25.29 (SD=2.60); range 20-29 | Instagram (photo-based platform) | Thin-ideal and sexually objectifying images via habitual/addictive Instagram use; photo-based appearance-focused content (other users' physical appearance). Instagram addiction measured via Bergen Facebook Addiction Scale adapted for Instagram (6 items: salience, mood modification, tolerance, conflict, withdrawal, relapse; 1-5) |

| ID | Author (Year) | Country | Study design | Recruitment | N | %F | Mean age | Platform(s) | Content/activity |
|---|---|---|---|---|---|---|---|---|---|
| S039 | Chang et al. (2019) | Singapore | Cross-sectional survey (paper and pencil); path analysis (AMOS 25; maximum likelihood); bootstrapping (5,000 resamples) for indirect effects | Secondary schools (3 schools: 2 co-educational, 1 single-sex female); purposive sampling; parental and participant consent required | 303 | 100% | range 12-16 | Instagram (image-focused social media platform) | Peer images on Instagram (photos of peers, edited selfies); three types of selfie practices: (1) photo browsing (viewing others' IG photos; 2 items; 1-6 frequency scale); (2) selfie posting (number of selfies posted per week; open-ended); (3) selfie editing (use of photo-editing apps, filters, multiple shots; 3 items; 1-5) |
| S040 | Meier & Gray (2014) | USA | Cross-sectional survey | Public middle/high school (New York State); voluntary sign-up during free periods | 103 | 100% | M=15.4; range 12-18 | Facebook | Appearance-focused photo content (photos of self and friends). Activity: passive (viewing friends' photos) and active (posting photos, updating profile photo, creating photo albums, tagging/untagging in photos, commenting on photos); operationalised as FB Appearance Exposure Score (photo subscale / total FB use) |
| S041 | Roberts et al. (2022) | USA | Cross-sectional survey; Structural Equation Modeling (SEM) | Large diverse suburban high school district (Florida); data collected during regular school hours via Character Lab Research Network (CLRN); pre-COVID (October 2019) | 543 | 100% | M=15.58 (SD=1.23); range 13-18 | Social media (unspecified; general social media appearance pressure measure); primarily Instagram and Snapchat referenced in literature review | Appearance-focused content (thinspiration, fitspiration, influencer/highly-edited images). Activity: general social media appearance pressure (perceived pressure to look thinner/more muscular); passive exposure implied; assessed via adapted SATAQ-4 social media subscale |
| S042 | Scully et al. (2023) | Ireland | Cross-sectional survey; serial multiple mediation analysis (Hayes PROCESS) | Three second-level schools in Ireland (one single-sex, one fee-paying, one urban); data collected during school hours (December 2015 - January 2016); parental consent obtained (59% response rate) | 210 | 100% | M=15.16 (SD=1.17); range 12-17 | Facebook | Appearance-focused photo content (photos of self and others, profile photos). Activity: passive and active appearance-related activities (viewing, updating profile photo, tagging); measured via Meier & Gray (2014) 8-item Photo Subscale (Appearance Exposure Score); social comparisons with photos of family, close friends, distal peers, and celebrities |
| S043 | Tiggemann et al. (2018) | Australia | Experimental (2x2 between-subjects: likes condition [low/high] x image type [thin-ideal/average]); laboratory setting | Flinders University (Adelaide); individual or small-group lab sessions; convenience sample of undergraduate students; course credit incentive | 220 | 100% | M=20.13 (SD=2.58); range 18-30 | Instagram (experimental stimuli presented in authentic Instagram frame on iPad) | Thin-ideal images (rated thinness 1-2/5) vs. average images (rated 3-4/5) of Caucasian women sourced from public Instagram profiles (#fashion, #beach, #plussize); images paired with low (1-10) or high (100-300) number of likes. Activity: passive viewing (15s per image); participants rated visual quality as an attention check |
| S044 | Brown & Tiggemann (2016) | Australia | Experimental (between-subjects, 3 conditions: celebrity images/peer images/travel images | Flinders University (Adelaide, South Australia); individual or small-group lab sessions; | 138 | 100% | M=20.10 (SD=2.61); range 18-30 | Instagram (images sourced from public Instagram profiles; presented on iPad | Attractive celebrity images (Kim Kardashian, Taylor Swift, Selena Gomez, Rihanna, Beyonce; top-10 most followed Instagram accounts) vs. equally attractive unknown peer images (#selfie hashtag, <200 followers) vs. travel |

| ID | Author (Year) | Country | Study design | Recruitment | N | %F | Mean age | Platform(s) | Content/activity |
|---|---|---|---|---|---|---|---|---|---|
| | | | [control]); laboratory setting | undergraduate convenience sample; course credit incentive | | | | without Instagram border) | images (control; #travel). Full-body to head-and-shoulder shots; 15 images per condition, 10s each. Activity: passive viewing with quality rating as attention check |
| S045 | Cohen et al. (2017) | Australia | Cross-sectional online survey (correlational) | Online recruitment via social media (university Facebook page and other social media outlets); voluntary participation; predominantly student sample (68% currently students) | 259 | 100% | M=22.97 (SD=3.25); range 18-29 | Facebook and Instagram | Facebook: appearance-focused photo activities (posting/viewing photos, updating profile photo, tagging) vs. general Facebook use; measured via Meier & Gray (2014) Facebook Questionnaire (FBQ, 23 items) and Appearance Exposure Score (AES = photo subscale / total FBQ). Instagram: following appearance-focused accounts (health & fitness: fitness bloggers, diet plans; celebrities: models, Kardashians) vs. appearance-neutral accounts (travel); measured via 3 single items (1-5 scale) |
| S046 | McComb & Mills (2021) | Canada | Experimental (2 conditions: forced upward body comparison to Instagram thin-ideal models vs. landscape painting control); laboratory; two-part design (trait measures online 1 week prior; in-lab manipulation) | York University (Toronto); undergraduate psychology participant pool; partial course credit incentive; recruited via online experiment management system (study titled "personality and social media") | 142 | 100% (self-identified) | range 18-24 | Instagram (images sourced from 2 public Instagram accounts of thin-ideal models with >11 million followers each; viewed on desktop computer in lab) | Thin-ideal images: 26 photos from 2 Caucasian Instagram models (bikini, fully clothed, face/shoulder close-ups; images with most likes selected); control: landscape paintings from public Instagram art account. Activity: forced active body comparison task (10 min; participants compared specific body parts and facial attractiveness to models, rated as much smaller/larger or more/less attractive); control performed art critique comparison task |
| S047 | Fioravanti et al. (2024) | Italy | Cross-sectional survey; Structural Equation Modeling (SEM) with serial mediation; case-control comparison (ED patients vs. controls) | ED patients: Eating Disorders Clinic, Florence University Hospital (first outpatient evaluation, DSM-5 diagnoses of AN, BN, or BED). Controls: university attendees and online (Facebook, Instagram, Twitter) via convenience snowball sampling. Inclusion criteria for all: female, age 18-65, active daily Instagram use | 386 (152 ED patients + 234 controls ) | 100% | M=26.04 (SD=6.73) | Instagram (daily use required for inclusion) | Idealized appearance-focused content (thin ideal, fitspiration, beauty-enhanced images); activity: general Instagram use measured as Problematic Instagram Use (PIU) - addictive/compulsive engagement pattern (salience, tolerance, mood modification, conflict, withdrawal, relapse); daily use in minutes also recorded |
| S048 | Vendemia & Fox (2024) | USA | Experimental (3x2 between-subjects online experiment: image type [sexualized | Large U.S. Midwestern university; undergraduate | 256 (279 completed) | 100% | M=20.06 (SD=1.57); range 18-29 | Instagram (stimuli: real images from pretested database presented as peer | Sexualized peer images (alluring/sexually suggestive poses, revealing clothing, e.g., duckface, cleavage shots) vs. non-sexualized peer images (fully clothed, non-suggestive; |

| ID | Author (Year) | Country | Study design | Recruitment | N | %F | Mean age | Platform(s) | Content/activity |
|---|---|---|---|---|---|---|---|---|---|
| | | | peers / non-sexualized peers/landscapes] x feedback condition [tagging / no tagging]) | courses; online study; course credit incentive; IRB approved | | | | Instagram posts; participants interacted via hashtag generation) | appearance-focused objectification) vs. landscape control. Images varied in attractiveness, body type, and skin color to reflect peer authenticity. Activity: passive viewing (13 images, embedded timer) OR active tagging (up to 5 hashtags per image, 65 total) |
| S049 | Samson & Zaitsoff (2023) | Canada | Experimental (3-condition between-subjects: general fitspiration [fit ideal only] / body transformation [before-after] / travel [control]); pre-post design with baseline measure | Western Canadian university; undergraduate psychology students (n=115, course credit) + university community members (n=46, gift card draw via email listserv and recruitment posters); two recruitment methods for larger/more diverse sample | 161 (after exclusions) | 100% (assigned female at birth) | M=20.00 (SD=2.86) | Instagram (images sourced from public Instagram accounts with >=100,000 followers; summer 2018) | General fitspiration (fit ideal only): fitness influencers in exercise clothing (sports bras, shorts; e.g., Krissy Cela). Body transformation: before-after pairs from fitness trainers featuring client/self transformations (e.g., Kayla Itsines, Anna Victoria). Control: travel photos. 24 images total per condition (16 experimental + 8 control for experimental groups); displayed 10s each with appearance-neutral T/F memory question. Activity: passive viewing |
| S050 | Rousseau (2024) | Belgium | Cross-sectional online survey | Social media recruitment | 345 | 100% | M=21.65 (SD=1.70); range 17-26 | Instagram | Body-positive content (posts challenging unrealistic appearance ideals, encouraging body acceptance, depicting diverse body types); thinspiration; fitspiration. Passive scrolling/viewing |
| S051 | Markey & Daniels (2022) | USA | Longitudinal online survey (2 time points, ~5 months apart) | Charter schools (5th-8th grade) + community (parenting/neighborhood Facebook groups) | 151 (T1) / 113 (T2) | 100% | M=12.06 (SD=1.22); range 10-15 | Instagram (56%), Snapchat (51%), YouTube (41%) | Appearance-focused content (beauty accounts, make-up, fashion, celebrities); communication-focused content (messages, photo posts). Passive viewing + active engagement (posting, following, messaging) |
| S052 | Prichard et al. (2023) | Australia | Experimental (between-subjects, 3 conditions: fashion Influencer, sexualized Influencer, control/fashion products) | Online recruitment (university research system + Facebook advertising) | 230 | 100% | M=20.46 (SD=2.31); range 17-25 | Instagram | Female Instagram Influencers: standard fashion images (fashionable attire) vs. sexualized images (lingerie/bikini, suggestive poses); control = fashion product images. Passive viewing (15 images per condition) |
| S053 | Patel et al. (2025) | USA | Experimental within-subjects (2 conditions: thinspiration videos vs. animal videos, counterbalanced order) | University psychology subject pool (lab-based) | 111 | ~100% (96.4% cis women, 3.6% non-binar | M=20.01 (SD=1.54) | TikTok | Thinspiration content (videos of thin individuals highlighting/glorifying thinness, body checks, dance, exercise, diet; hashtags #bikini, #bodycheck); control = animal videos. Passive viewing (5-min compilations, ~16 videos each) |

| ID | Author (Year) | Country | Study design | Recruitment | N | %F y/trans) | Mean age | Platform(s) | Content/activity |
|---|---|---|---|---|---|---|---|---|---|
| S054 | Tiggemann & Barbato (2018) | Australia | Experimental between-subjects (2 conditions: appearance comments vs. place/control comments) | University participant pool (lab-based, individually or small groups) | 128 | 100% | M=20.12 (SD=2.46); range 18-30 | Instagram | Attractive women in travel/nature locations; experimental manipulation: positive appearance comments (e.g., 'Great legs', 'You look amazing') vs. place comments (e.g., 'Great beach'). Passive browsing (15 images, 15 sec each) |
| S055 | Pryde & Prichard (2022) | Australia | Experimental between-subjects (2 conditions: fitspiration TikTok videos vs. art TikTok control videos) | University psychology pool + online (Facebook groups, university volunteer webpage) | 120 | 100% | M=21.06 (SD=2.40); range 17-25 | TikTok | Fitspiration videos (women exercising or posing in activewear, fit/toned body; hashtag #fitspo; 10 min of content); control = art TikTok videos (individuals creating/showcasing artwork; hashtag #art). Passive viewing |
| S056 | Becker et al. (2025) | Netherlands/Germany/Belgium/USA (multinational; primarily Netherlands) | Cross-sectional online survey | Online convenience sampling (physical boards, SNS, online recruitment platforms) | 454 | 100% | M=22.65 (SD=3.57); range 18-35 | Instagram | Appearance-ideal content (idealized physical beauty per cultural standards: low body fat, thin waist, hourglass figure) and body-positive content (diverse bodies, acceptance/respect for all bodies). Engagement: viewing, liking, commenting, sharing, posting |
| S057 | Engeln et al. (2020) | USA | Experimental between-subjects (3 conditions: Instagram, Facebook, control/Bejeweled matching game); 2 (time) x 3 (condition) mixed design | University (introductory psychology pool + campus library/residence halls) | 308 | 100% | M=19.40 (SD=1.23); range 18-26 | Instagram; Facebook (comparison condition) | Real-world social media feeds (Instagram and Facebook); participants used their own accounts as they normally would for 7 minutes (posting, liking, commenting, browsing allowed). Control = Bejeweled matching game (no appearance content) |
| S058 | Anixiadis et al. (2019) | UK/Europe/North America (multinational; 58.7% UK) | Experimental between-subjects (2 conditions: thin-ideal Instagram images vs. control scenery images) + qualitative thought-elicitation (thought listing) | Online crowdsourcing (Prolific Academic) | 126 (exp); 91 (thought-listing subsample) | 100% | M=23.28 (SD=2.58); range 18-29 | Instagram (images sourced from Instagram) | Thin-ideal images: 9 images of thin, attractive Caucasian young women (18-25) in minimal apparel (bathing suits), peer-posted style (non-professional production). Control: 9 scenery images. Passive viewing + thought listing after each of 6 images |

***Note.*** Studies are identified by Study ID (S001–S058) and first author with year of publication. Full citations are listed in the reference list of the main manuscript.
