# Supplementary material for: Bodies on Display: A Scoping Review of Appearance-Focused Social Media, Body Image, Self-Esteem, and Psychological Wellbeing in Adolescent Girls and Young Women
Source: Eur J Investig Health Psychol Educ. 2026 Jul 23;16(8):108. doi: 10.3390/ejihpe16080108 (PMC13512515; doi:10.3390/ejihpe16080108)
Supplement: Supplementary file 1 [file ejihpe-16-00108-s001.zip › Table_S3_Outcomes_Mechanisms.pdf]

Table S3. Outcomes, instruments, mechanisms, and theoretical frameworks of the 58 included studies

Bodies on Display: A Scoping Review of Appearance-Focused Social Media, Body Image, Self-Esteem, and Psychological Wellbeing in Adolescent Girls and Young Women

ID	Author (Year)	Body image constructs	Self-esteem/wellbeing	Measurement instruments	Mediators	Moderators / protective	Framework
S001	Lee & Lee (2021)	Body satisfaction		SNS Appearance-Related Photo Activity scale (Lee & Lee, 2017; 6 items); SATAQ-4R Internalization-General Attractiveness subscale (Schaefer et al., 2017; 7 items); Appearance comparison scale (Hendrickse et al., 2017; 3 items); MBSRQ-AE (Cash, 2000; 7 items); Photo-editing behavior: single-item frequency measure	Internalization of body ideals; appearance comparison	Photo-editing behavior (moderates paths: photo activity → internalization; internalization → appearance comparison)	Tripartite influence model; social comparison theory; objectification theory
S002	Fardouly & Holland (2018)	State body dissatisfaction; positive impressions of the target	negative mood	State body dissatisfaction: VAS (4 items; α=.84); State negative mood: VAS (5 items; α=.88); Positive impressions: VAS (8 traits; α=.87); SATAQ-4 Internalization-Thin/Low Body Fat subscale (Schaefer et al., 2015; 5 items); PACS-R (Schaefer & Thompson, 2014; 11 items)	None tested	Thin ideal internalization (SATAQ-4; n.s.); appearance comparison tendency (PACS-R; significant for body dissatisfaction and impressions)	Tripartite influence model; social comparison theory
S003	Roorda et al. (2026)	Body image; body dissatisfaction; dieting intentions		BISS (Cash et al., 2002; 6 items; α=0.83/0.87); Body Dissatisfaction VAS (single item, 0–100); Dieting Intentions VAS (3 items, developed for the study; α=0.87)	None tested	None formally tested (thin-ideal internalization and BMI suggested as future moderators)	Social comparison theory; sociocultural theory of body image
S004	Yue & Tang (2024)	Weight body-esteem; appearance body-esteem		Idealized content exposure: 8-item scale developed for the study (α=.92); Non-idealized content exposure: 8-item scale (de Lenne et al., 2022; α=.80); UPACS (O'Brien et al., 2009; 10 items; α=.94); DACS (O'Brien et al., 2009; 8 items; α=.94); Body Esteem Scale – weight (6 items; α=.93) and appearance (7 items; α=.90) subscales (Mendelson et al., 2001); SOQ (Noll & Fredrickson, 1998)	Upward appearance comparison (UPACS); downward appearance comparison (DACS)	Trait self-objectification (SOQ; significant moderator of path idealized content → upward comparison)	Tripartite influence model; social comparison theory; objectification theory
S005	Cohen & Blaszczynski (2015)	State body image dissatisfaction (BID-change); eating disorder risk		BASS/MBSRQ (Cash, 2000; 9 items; α=.789); Extent Thoughts Questionnaire (Bessenoff, 2006; 3 items; α=.94) – appearance comparison; SATAQ-3 Pressure subscale (Thompson et al., 2004; 7 items; α=.83); Rosenberg Self-Esteem Scale (1965; 10 items; α=.88); EAT-26 (Garner et al., 1982; 26 items; α=.870); Facebook use:	None formally tested (appearance comparison as key predictor of BID-change)	Type of exposure (Facebook vs conventional media; n.s.); thin-ideal internalization (covariate); self-esteem (covariate)	Social comparison theory; tripartite influence model

ID	Author (Year)	Body image constructs	Self-esteem/wellbeing	Measurement instruments	Mediators	Moderators / protective	Framework
				self-report composite (hours × accesses/day × days/week)			
S006	Fardouly et al. (2017)	Appearance satisfaction; thoughts of dieting/exercising; diet and exercise behavior	mood	EDI Body Dissatisfaction subscale (Garner et al., 1983; 9 items; α=.86) – trait measure; State appearance satisfaction: SSES Appearance subscale (Heatherton & Polivy, 1991; 6 items; α=.87); Mood: 10-item scale (positive/negative words; α=.73); Thoughts of dieting/exercising: 2 items adapted from EDE-Q (Fairburn & Beglin, 1994; α=.78); Diet and exercise behavior: 2 dichotomous items	None formally tested	Trait body dissatisfaction (EDI; entered as Level-2 moderator in HLM; n.s. for all context interactions)	Social comparison theory; sociocultural model of body image
S007	Zhong (2022)	body image disturbance	Appearance anxiety	Self-made questionnaire (Xiaohongshu use frequency and duration); Appearance Anxiety Scale-Brief Version (revised from Dion et al., 1990; 6 items; α=.805); SATAQ-3 – ideal beauty internalization (Thompson et al., 2004, revised by Liu Daqing, 2009; 5 items; α=.803); Modified PACS – appearance comparison on Xiaohongshu (Thompson et al., 1999; 7 items; α=.778)	Internalization of ideal beauty (→ body image disturbance; tested separately, not as formal mediation)	None formally tested	Sociocultural theory; tripartite influence model; social comparison theory
S008	Kim (2018)	Weight satisfaction; drive for thinness		Facebook use for profile maintenance: 6-item scale (α=.92); Facebook use for social grooming: 7-item scale (Kim & Chock, 2015; α=.93); Appearance Social Comparison: adapted PACS (Thompson et al., 1991; 6 items; α=.97) – peer-focused; BESAA Weight Satisfaction subscale (Mendelson et al., 2001; 8 items; α=.86); EDI Drive for Thinness subscale (Garner et al., 1983; 7 items; α=.89)	Appearance comparison with peers on Facebook (mediates: profile maintenance → body image; social grooming → body image)	None tested	Social comparison theory
S009	Kowal (2026)	Mirror gaze duration; mirror self-fixation count; self-modification count; self-assessed physical attractiveness	self-esteem	Behavioral coding via BORIS software v7.13.8 (3 blind coders; ICC: gaze alpha=.95, modifications alpha=.95, self-fixations alpha=.90); Rosenberg Self-Esteem Scale (alpha=.89); single-item physical attractiveness scale (11-point Likert)	None tested	Age, BMI, self-esteem, time on social media, physical attractiveness (covariates in regression); no significant moderation detected	Social Comparison Theory (Festinger, 1954); Objectification Theory / Self-Objectification Theory (McKinley & Hyde, 1996)

ID	Author (Year)	Body image constructs	Self-esteem/wellbeing	Measurement instruments	Mediators	Moderators / protective	Framework
S010	Etherson (2022)	body appreciation; appearance-focused upward social comparison	Depressive symptoms	Big Three Perfectionism Scale (BTPS; rigid perfectionism alpha=.91, self-critical perfectionism alpha=.94); Body Appreciation Scale-2 (BAS-2, 4 items; alpha=.84-.95); 3-item depressive symptoms scale (alpha=.86-.93); single-item upward social comparison (direction + context)	None tested (perfectionism measured as between-person predictor, not mediator)	Self-critical perfectionism (significant moderator: amplifies depressive symptoms and reduces body appreciation under upward social comparison); rigid perfectionism (tested but non-significant moderator)	Social Comparison Theory (Festinger, 1954); Specific Vulnerability Hypothesis (Hewitt & Flett, 1993)
S011	Tiggemann (2015)	body dissatisfaction; state appearance self-esteem (appearance subscale); state appearance comparison; inspirational goals (fitness, healthy eating, travel)	Negative mood	Visual Analogue Scales (VAS; mood alpha=.75, body dissatisfaction alpha=.86); State Self-Esteem Scale (SSES; appearance subscale alpha=.87); State Appearance Comparison Scale (3 items, alpha=.91); Physical Appearance Comparison Scale (PACS; trait, alpha=.72)	State appearance comparison (significant full mediator of body dissatisfaction and appearance self-esteem; partial mediator of negative mood)	Trait appearance comparison (tested as moderator via hierarchical regression; non-significant for all outcomes)	Social Comparison Theory (Festinger, 1954); Sociocultural Model (Thompson et al., 1999; Tiggemann, 2011); Objectification Theory (Fredrickson & Roberts, 1997)
S012	Seekis (2023)	Face-related appearance shame; state self-compassion; upward appearance comparisons; appearance thoughts	appearance anxiety; negative mood	Phenomenological Body Shame Scale-Revised (PBSS-R, 8 items; alpha=.92); Physical Appearance State and Trait Anxiety Scale - state subscale (PASTAS, 8 facial items; alpha=.77-.84); State Self-Compassion Scale - Short Form (SSCS-S, 6 items; alpha=.67-.77); 4-item VAS negative mood (alpha=.74-.75); State Appearance Comparison Scale (SACS, modified for TikTok/upward direction; Spearman-Brown predicted reliability=0.95)	None tested	Self-compassion content (protective factor: beauty group showed lower self-compassion and higher shame/anxiety than self-compassion and control groups; self-compassion group showed higher self-compassion than control)	Objectification Theory (Fredrickson & Roberts, 1997); Social Comparison Theory (Festinger, 1954); Tripartite Influence Model (Thompson et al., 1999)
S013	Galway (2025)	Body-related shame; body-related guilt; body-related envy; intentions to change diet; state upward appearance comparisons		Body and Appearance Self-Conscious Emotions Scale (BASES; shame alpha=.94-.96, guilt alpha=.93-.95) adapted to VAS (0-100); Body-Related Envy Scale - appearance subscale (4 items, VAS; alpha=.91-.92); 2-item state upward appearance comparisons (SACS modified; alpha=.90); Diet and Exercise Intentions Questionnaire - diet subscale (8 items; alpha=.93); Self-Compassion Scale - Short Form (SCS-SF, 12 items; alpha=.90)	Upward appearance comparisons (significant mediator of WIEIAD vs. travel on all outcomes: shame, guilt, envy, and diet intentions)	Trait self-compassion (tested as moderator via PROCESS macro 8; non-significant for all outcomes and for appearance comparisons)	Social Comparison Theory (Festinger, 1954); Sociocultural Theory; Self-Conscious Emotions framework (Tangney et al., 2007)
S014	Fioravanti (2023)	body satisfaction; state appearance comparison (daily, over 28 days)	Positive mood; negative mood	Computer-based Visual Analogue Scale (C-VAS, 1-10): positive mood (happy + confident, alpha=.72),	None tested	Body-positive content (protective factor: highest growth in positive mood	Social Comparison Theory (Festinger, 1954);

ID	Author (Year)	Body image constructs	Self-esteem/wellbeing	Measurement instruments	Mediators	Moderators / protective	Framework
				negative mood (depressed + anxious + anger, alpha=.81), body satisfaction (4 items, alpha=.97); State Appearance Comparison Scale (SACS, 3 items, alpha=.95); Physical Appearance Comparison Scale (PACS, trait, alpha=.73); MBSRQ-AS (trait body image, baseline)		and body satisfaction); fitspiration (risk factor: highest growth in negative mood and appearance comparison)	Objectification Theory (Tiggemann, 2011); Positive Body Image framework (Tylka & Wood-Barcalow, 2015)
S015	Walker (2015)	Disordered eating (EDE-Q4 global score); Facebook intensity; online physical appearance comparison; online fat talk		Eating Disorder Examination Questionnaire-4 (EDE-Q4, 36 items; alpha=.92); Facebook Intensity Scale (8 items; alpha=.87); Online Physical Appearance Comparison Scale (modified PACS; alpha=.78); Online Fat Talk Scale (adapted, 9 scenarios; alpha=.91); BMI; BDI-II (alpha=.93); STAI-trait (alpha=.94); MPS concern over mistakes (alpha=.92); UPPS Negative Urgency (alpha=.88); GSE (alpha=.89)	Online physical appearance comparison (significant mediator between Facebook intensity and disordered eating); online fat talk (direct predictor; mediation path non-significant)	BMI, depression, trait anxiety, perfectionism, impulsivity, self-efficacy (covariates); Facebook intensity (protective when appearance comparison controlled)	Social Comparison Theory (Festinger, 1954); Sociocultural Model of disordered eating
S016	Belmonte (2024)	Endorsement of voluptuous body ideal; appearance-related comparisons; perception of Instagram as source of pressure about appearance ideals; perception of Instagram as source of information about appearance ideals		Instagram Intensity Scale (modified Facebook Intensity Scale, 6 items; alpha=.89); SATAQ-3 Pressure subscale adapted for Instagram (5 items; alpha=.90); SATAQ-3 Information subscale adapted for Instagram (5 items; alpha=.84); PACS-R (11 items; alpha=.97); Ideal-Body Stereotype Scale-Revised adapted for voluptuous body (4 items; alpha=.85)	Perception of Instagram as source of pressure (significant M1) and appearance-related comparisons (significant M2) serially mediate the path from Instagram intensity to voluptuous body endorsement; perception of Instagram as source of information (significant M1) and appearance comparisons (M2) also form a significant serial path	Instagram intensity (critical predictor over frequency of use); frequency of use (non-significant predictor in serial paths)	Sociocultural Model (Thompson et al., 1999); Social Comparison Theory (Festinger, 1954)
S017	Dajches (2025)	Body appreciation; body dissatisfaction; acceptance of cosmetic surgery		Body Appreciation Scale-2 (BAS-2, 10 items; alpha=.95); Body Image Ideals Questionnaire (10 physical characteristics, discrepancy x importance; alpha=.83-.85); Acceptance of Cosmetic Surgery Scale (15 items; alpha=.90); single-item appearance comment frequency; 5-point ordinal scales for celebrity and health/fitness account following	Body dissatisfaction (significant mediator between following celebrity TikTok accounts and acceptance of cosmetic surgery); body appreciation (non-significant mediator in all paths)	Leaving appearance-related comments (directly associated with higher body appreciation and higher cosmetic surgery acceptance); following health/fitness accounts (directly associated with cosmetic surgery acceptance, not mediated); TikTok daily use (controlled)	Social Comparison Theory (Festinger, 1954); Tripartite Influence Model (Thompson et al., 1999)

ID	Author (Year)	Body image constructs	Self-esteem/wellbeing	Measurement instruments	Mediators	Moderators / protective	Framework
S018	Chansiri et al. (2020)	Body dissatisfaction (state); appearance comparison (state); ideal body internalization (state); self-objectification (state); sexual self-efficacy; sexual initiation assertiveness; sexual self-consciousness		State body dissatisfaction: 2 VAS (100mm; Tiggemann & Zaccardo, 2015); State appearance comparison: 3 items from TACS (Tiggemann & McGill, 2004; α=.91); State ideal body internalization: SATAQ-4 (Schaefer et al., 2016; 9 items; α=.93); State self-objectification: SOQ (Noll & Fredrickson, 1998; α=.78); Sexual self-efficacy: SRBBS (Basen-Engquist et al., 1999; 9 items; α=.79); Sexual initiation assertiveness: Initiation subscale SAS (Morokoff et al., 1997; 6 items; α=.81); Sexual self-consciousness: Sexual-Self Consciousness Scales (Van Lankveld et al., 2008; 12 items; α=.80)	State appearance comparison → state body dissatisfaction (H1, supported); state ideal body internalization → state self-objectification (H2, not supported); serial chain: appearance comparison → self-objectification → body dissatisfaction (RQ2, significant for sexual self-efficacy and sexual self-consciousness)	No moderators formally tested. Confounders controlled: ethnicity (White/non-White), romantic relationship status (yes/no), eating disorder history (yes/no). No protective factors tested.	Sociocultural Theory / Tripartite Influence Model (Thompson et al., 1999); Objectification Theory (Fredrickson & Roberts, 1997); novel combined model integrating sociocultural and objectification theory components
S019	Flynn & Newman (2023)	State body satisfaction; state self-objectification; inspiration to improve fitness; inspiration to eat healthily; state appearance comparison; overall image inspiration rating	State negative mood	State body satisfaction: 3 VAS items (overall appearance, weight, body shape; based on Slater et al., 2017; αpre=.88, αpost=.93); State negative mood: 5 VAS items (anxiety, depression, happiness [rev], anger, confidence [rev]; cf. Tiggemann & Zaccardo, 2015; αpre=.74, αpost=.78); State self-objectification: 8-item adapted Body Surveillance subscale of OBCS (McKinley & Hyde, 1996), state version, 7-point Likert; αpre=.74, αpost=.77); State appearance comparison: 3 VAS items from State Appearance Comparison Scale (Tiggemann & McGill, 2004; α=.95); Inspiration ratings: VAS items (improve fitness, eat healthily, travel; pre & post)	State appearance comparison mediated the relationship between image condition and: (a) state negative mood change (significant for both experimental conditions vs. control); (b) state self-objectification change (significant for both conditions vs. control). Mediation via appearance comparison for body satisfaction change was NOT significant.	None formally tested. Note: viewing the same bodies without weight loss context (non-side-by-side condition) increased body satisfaction — may function as a partial protective framing effect, but not tested as a moderator.	Social Comparison Theory (Festinger, 1954); Objectification Theory / Self-Objectification Theory (Fredrickson & Roberts, 1997)
S020	Daniels & Robnett (2021)	Body surveillance; body shame; math-science (MS) self-expectancy; MS task value; MS future plans; MS perceived cost		Appearance-focused social media behaviors: photo subscale of FBQ (Meier & Gray, 2014; 8 items; α=.90); Body surveillance: OBC-Youth body surveillance subscale (Lindberg et al., 2006; 4 items; α=.87); Body shame: OBC-Youth body shame subscale (Lindberg et al., 2006; 5 items; α=.90); MS self-expectancy, task value, cost: EVC scale (Kosovich et al., 2014; 3–4	Body shame (not body surveillance) partially mediated the association between appearance-focused social media behaviors and: (a) MS self-expectancy (\u03b2=\u2013.09, p<.01); (b) MS cost (\u03b2=.16, p<.001). MS task value and MS	No moderators tested. MS grade (self-reported) controlled as covariate. Ethnicity (African American vs. European American): no significant multivariate differences in body image or academic attitude variables.	Objectified Body Consciousness (OBC) framework (McKinley & Hyde, 1996); Objectification Theory (Fredrickson & Roberts, 1997); Expectancy-Value-Cost Theory (Eccles & Wigfield, 1995,

ID	Author (Year)	Body image constructs	Self-esteem/wellbeing	Measurement instruments	Mediators	Moderators / protective	Framework
				items/subscale; α=.86–.94); MS future plans: scale from Kosovich et al. (2014; 3 items; α=.87–.91). All rated on 1–6 or 1–7 Likert scales.	future plans were not significantly associated with OBC mediators and were dropped from mediation analyses. MS grade controlled throughout.		2002, 2020; Kosovich et al., 2014)
S021	Fardouly et al. (2018)	Body dissatisfaction; drive for thinness; self-objectification; internalization of beauty ideal; appearance comparison tendency (general); appearance comparisons to specific Instagram targets (family, friends, acquaintances, strangers, celebrities, self, fitspiration women)		Instagram use: 2 custom items (z-scored and averaged); Fitspiration viewing: 1-item (1–5); Comparison to targets: custom 5-point items; Internalization: SATAQ-3 Internalization-General (Thompson et al., 2004; 9 items; α=.95); Appearance comparison tendency: UDACS (O'Brien et al., 2009; 18 items; α=.95); Body dissatisfaction: EDI-BD (Garner et al., 1983; 9 items; α=.90); Drive for thinness: EDI-DFT (7 items; α=.90); Self-objectification: SOQ (Noll & Fredrickson, 1998)	Overall Instagram use → self-objectification: internalization (significant) and comparisons to celebrities (significant); acquaintances and strangers: n.s. Fitspiration viewing → body dissatisfaction & drive for thinness: internalization (significant), appearance comparison tendency (significant), comparisons to fitspiration women (significant). Internalization was a stronger mediator for drive for thinness.	Sample (MTurk vs. student) tested as moderator: pattern of associations did not vary significantly. No protective factors tested.	Sociocultural model of body image (Thompson et al., 1999; Van den Berg et al., 2002); Social Comparison Theory (Festinger, 1954); Objectification Theory (Fredrickson & Roberts, 1997); Tripartite Influence Model
S022	Fardouly et al. (2015)	state body dissatisfaction; weight & shape appearance discrepancy; face, hair & skin appearance discrepancy	State negative mood	State negative mood: VAS (5 items; α=.71); State body dissatisfaction: VAS (3 items; α=.74); Appearance discrepancy: state Self-Discrepancy Index (SDI; Dittmar et al., 1996; scores 0–75); Trait appearance comparison tendency: UPACS/DACS (O'Brien et al., 2009; 18 items; α=.92)	No formal mediation tested. Appearance comparison tendency tested as MODERATOR only.	Trait appearance comparison tendency (UPACS/DACS) moderated Facebook → face/hair/skin discrepancy: significant for high comparison tendency (β=.47, p=.007), not for low (β=–.12, p=.419). Did not moderate mood, body dissatisfaction, or weight/shape discrepancy. Low comparison tendency: protective factor against face/hair/skin discrepancy on Facebook.	Social Comparison Theory (Festinger, 1954); Sociocultural Model of body image (Keery et al., 2004; van den Berg et al., 2002)
S023	Slater & Tiggemann (2015)	Self-objectification; self-surveillance; body shame; disordered eating (drive for thinness)		Self-objectification: SOQ (Noll & Fredrickson, 1998; ipsative, −25 to +25); Self-surveillance: OBC-Y Surveillance subscale (Lindberg et	Body shame mediated: (a) self-objectification → disordered eating (β=.172, 95% CI [.180,	No moderators formally tested. Extracurricular activities (all types) NOT significantly associated	Objectification Theory (Fredrickson & Roberts, 1997); Sociocultural

ID	Author (Year)	Body image constructs	Self-esteem/wellbeing	Measurement instruments	Mediators	Moderators / protective	Framework
				al., 2006; 4 items; α=.90); Body shame: OBC-Y Body Shame subscale (5 items; α=.87); Disordered eating: EDI-DFT (Garner et al., 1983; 7 items; α=.91); Media/extracurricular/comments: custom items	.230]); (b) self-surveillance → disordered eating (β=.195, 95% CI [.262, .342]). In path model: magazines & SNS → self-objectification → self-surveillance → body shame → disordered eating.	with self-objectification cross-sectionally. Longitudinal protective effect of organized sport documented separately (Slater & Tiggemann, 2012).	Model of body image
S024	Zhou et al. (2024)	Body dissatisfaction; cosmetic surgery consideration; internalization of beauty ideals; peer appearance comparisons; beauty social media attention		Beauty social media attention: 5-item scale (Seekis et al., 2020); Peer appearance comparisons: 4-item scale (Jones, 2001); Body dissatisfaction: 5-item scale (Garner et al., 1983); Cosmetic surgery consideration: 4-item scale (Swami et al., 2008); Internalization: 4-item scale (Thompson et al., 1999); All items 5-point Likert	Peer appearance comparisons mediate peer conversations to body dissatisfaction and cosmetic surgery consideration (significant); Internalization of beauty ideals mediates beauty social media attention to body dissatisfaction (significant)	None tested	None identified
S025	Zulec Ivankovic et al. (2024)	Appearance satisfaction; appearance comparison with Instagram content; internalization of beauty ideals; media pressure		Appearance satisfaction: BESAA BE-Appearance subscale (Mendelson et al., 2001; 7 items); Appearance comparison with Instagram: PACS-R adapted (Schaefer & Thompson, 2014; 10 items); Internalization: SATAQ-4 (Schaefer et al., 2015; 2 subscales: thin/low body fat, athletic/muscular); Media pressure: SATAQ-4 Pressures subscale; Instagram use: frequency/time custom items	Appearance comparison with Instagram mediates media pressure to appearance satisfaction (significant); Internalization mediates media pressure to appearance satisfaction via appearance comparison (partial mediation in path model)	None tested	None identified
S026	Ferdousi et al. (2023)	Willingness to act to be thinner (diet/exercise); willingness to act to be curvier; thin/curvy body preference; internalization of thin/curvy ideal		Thin/curvy model exposure: 2 original items (1-5); Thin/curvy body preference: 2 original items (1-5); Internalization: 8 items adapted from SATAQ-3 (Thompson et al., 2004; thin and curvy subscales); Willingness to act to be thinner/curvier: original items (1-5); BMI: self-reported height/weight	Model 1 (simple): thin/curvy body preference mediates exposure to willingness to act to be thinner/curvier (significant). Model 2 (serial): thin/curvy body preference to internalization to willingness (significant serial mediation)	BMI (controlled covariate)	None identified
S027	Livingston et al. (2020)	Body dissatisfaction (state); perceived realism of social	Negative mood (state)	Body dissatisfaction and mood: visual analogue scales (0-100; Heinberg & Thompson, 1995	Appearance comparison tested as potential mechanism (no	Ethnicity noted as potential moderator (not formally tested); degree	Self-disclaimer captions (tested but found

ID	Author (Year)	Body image constructs	Self-esteem/wellbeing	Measurement instruments	Mediators	Moderators / protective	Framework
		media images; appearance comparison		adapted); Perceived realism: custom scale (1-7); Appearance comparison: custom items (1-7); Instagram image exposure: experimental manipulation (attractive/edited vs. body-positive vs. neutral)	significant mediation found); perceived realism tested as covariate	of own image editing (not formally tested)	ineffective); media literacy (suggested for future); body-positive content (partial protective effect on mood but not body dissatisfaction)
S028	Hogue & Mills (2019)	State body image dissatisfaction (overall appearance dissatisfaction + body dissatisfaction composite); appearance comparison (upward social comparison)	State negative mood	State Body Image Scale (SBIS): average of VAS-Overall Appearance Dissatisfaction + VAS-Body Dissatisfaction (Heinberg & Thompson, 1995); State Mood Scale: adapted PANAS negative affect subscale (5 items); Upward social comparison: custom item (1-7); Social media use: custom (time spent on SM in last hour; types of SM used)	Upward social comparison tested as theoretical mechanism (not formally measured as mediator in statistical model); peer vs. family condition as key manipulated variable	None formally tested; peer vs. family social media condition as key moderating variable	Engaging with family member social media (no negative effect on body image; protective relative to peer condition)
S029	Hai & Yang (2022)	body area satisfaction; overweight preoccupation; social network site appearance comparison (SNSAC)	Anxiety; self-esteem	SNSACS (Fardouly & Vartanian, 2015; 3 items, 5-point Likert; Chinese version validated); Body area satisfaction: MBSRQ-AS subscale (Cash, 2000; 9 items); Overweight preoccupation: MBSRQ-AS subscale (4 items); Self-esteem: Rosenberg Self-Esteem Scale (1965; 10 items); Anxiety: GAD-7 (Spitzer et al., 2006; 7 items)	SNSAC to body area satisfaction to self-esteem to anxiety (chain mediation; all steps significant); SNSAC to overweight preoccupation to self-esteem to anxiety (chain mediation; significant)	None tested	Higher body area satisfaction and higher self-esteem protective against anxiety in the chain mediation
S030	Choukas-Bradley et al. (2019)	ASMC frequency; body surveillance (self-objectification); body comparison; body esteem	depressive symptoms	ASMC: 4-item scale developed for study (1-7 Likert; alpha=0.83); Body Surveillance: OBC-SS (McKinley & Hyde, 1996; 8 items); Body Comparison: IBCS (Fisher et al., 2002; 8 items); Body Esteem: BES (Franzoi & Shields, 1984; 35 items); Depressive Symptoms: CES-D (Radloff, 1977; 20 items); Time on social media: custom item (hrs/day)	Body surveillance and body comparison tested as parallel outcomes of ASMC (Path 1 model); body esteem and depressive symptoms as downstream outcomes (Path 2 model); indirect paths ASMC to body esteem/depression via body surveillance/comparison (significant)	Time spent on social media (controlled in both path models)	Higher body esteem protective against depressive symptoms; social media literacy (suggested future protective factor)
S031	Fardouly & Vartanian (2015)	Body dissatisfaction; drive for thinness; Facebook appearance comparisons (general + target-specific: celebrities, distant peers, close friends, family)		Facebook usage: 2-item composite (frequency of checking + daily time, standardized and averaged); Body dissatisfaction: BSQ-8C (Cooper et al., 1987; adapted); Drive for thinness: EDI-2 (Garner, 1991; 7 items); Facebook	Appearance comparisons in general mediate Facebook usage to body dissatisfaction (significant); Appearance comparisons to celebrities mediate	None formally tested	None identified

ID	Author (Year)	Body image constructs	Self-esteem/wellbeing	Measurement instruments	Mediators	Moderators / protective	Framework
				appearance comparisons: SPSCS adapted (Morrison et al., 2004; general + target-specific subscales)	Facebook usage to drive for thinness (significant); Close friend comparisons not a significant mediator		
S032	Seekis & Lawrence (2023)	Functionality appreciation (state); body satisfaction (state); internalization of thin ideal (state)	positive mood (state)	Functionality Appreciation Scale (FAS; Alleva et al., 2017; 7 items, VAS 0-100); Body Satisfaction Scale (BSS; Slade et al., 1990; 16 items, VAS 0-100); Positive Mood (PANAS positive affect subscale; 10 items, VAS 0-100); Internalization of Thin Ideal: custom VAS item; Pre-test scores as covariates in ANCOVA	None formally tested as mediators; condition (body neutrality vs. thin ideal vs. neutral) as IV; pre-test scores as covariates	Pre-test scores (as covariate in ANCOVA)	Body neutrality TikTok content (significant protective factor: higher functionality appreciation, body satisfaction, positive mood vs. thin ideal condition)
S033	Yang et al. (2020)	Body esteem (global; 3 subscales: appearance, attribution, weight)		Body Esteem Scale for Adolescents and Adults (BESA; Mendelson et al., 2001; 21 items); Social Appearance Anxiety Scale (SAAS; Hart et al., 2008; 16 items); SATAQ-3 General Internalization subscale (9 items); PACS-R (Schaefer & Thompson, 2014; 10 items); MTUAS for social media use; Dieting Beliefs Scale internal locus of control subscale (covariate); PROCESS macro (Hayes, 2009)	Serial mediation: social media/smartphone use to cognitive internalization of thin ideals (SATAQ-3) to appearance comparison (PACS-R) to social appearance anxiety (SAAS) to body esteem. Heavy social media use (>3h/day) and excessive smartphone use (>4h/day) showed significant serial indirect effects. Website browsing, TV shows, and music listening showed significant indirect effects independent of social media	Moderators: none formally tested. BMI, age, household income, and internal locus of control over weight used as covariates. Protective factors: none explicitly identified	Tripartite Influence Model (Thompson et al., 1999); Social Comparison Theory (Festinger, 1954); Cognitive-affective framework integrating internalization, social comparison, and social appearance anxiety
S034	Seekis et al. (2020)	Drive for thinness; body dissatisfaction		Appearance-Related SNS Scale (developed for study; 3 subscales: CFB 6 items, fitspiration 5 items, ILC 6 items; 1-5); Upward Physical Appearance Comparison Scale (UPACS; O'Brien et al., 2009; 10 items; 1-5); Body Surveillance subscale - Objectified Body Consciousness Scale (OBC-SS; McKinley & Hyde, 1996; 8 items; 1-7); Social Appearance Anxiety Scale (SAAS; Hart et al., 2008; 16 items; 1-5); Drive for Thinness subscale - EDI-3 (Garner, 2004; 7 items; 1-6);	Serial mediation (5,000 bootstrapped samples): CFB and ILC had significant indirect effects on drive for thinness and body dissatisfaction via upward appearance comparison (UPACS) to body surveillance (OBC-SS) to social appearance anxiety (SAAS). Fitspiration-related content had NO significant indirect	Moderators: none formally tested. BMI included as covariate (correlated only with body dissatisfaction and drive for thinness). Protective factors: none identified; media/social media literacy programs suggested as future protective interventions	Objectification Theory (Fredrickson & Roberts, 1997); Social Comparison Theory (Festinger, 1954); Integrated Objectification-Social Comparison Model (extending Tylka & Sabik, 2010; Fitzsimmons-Craft et al., 2014; Lindner et al., 2012)

ID	Author (Year)	Body image constructs	Self-esteem/wellbeing	Measurement instruments	Mediators	Moderators / protective	Framework
				Body Dissatisfaction subscale - EDI-3 (10 items; 1-6)	effects (non-significant path fitspiration to body surveillance); fitspiration showed only significant DIRECT effects on both drive for thinness and body dissatisfaction		
S035	Fardouly et al. (2015)	Self-objectification		Self-Objectification Questionnaire (SOQ; Noll & Fredrickson, 1998; 10-item ranking: 5 appearance attributes vs. 5 competence attributes; score range -25 to +25); Media usage: custom items for each medium (time per day or frequency); Upward and Downward Appearance Comparison Scale (O'Brien et al., 2009; 18 items; 1-5); Appearance comparisons to specific Facebook target groups: custom single items per target (self, family, close friends, distant peers, celebrities; 1-5)	General appearance comparison tendency mediated: (1) magazine usage to self-objectification (significant; 95% CI [0.24, 2.66]); (2) Facebook usage to self-objectification (significant; 95% CI [0.30, 2.01]). Target-specific mediators of Facebook to self-objectification: comparisons to close friends (significant; 95% CI [0.23, 1.80]); comparisons to distant peers (significant; 95% CI [0.60, 2.35]); comparisons to self (non-significant). In multiple mediation, only distant peers remained significant (95% CI [0.03, 1.92]). TV, Internet, and music video usage NOT associated with self-objectification	Moderators: none formally tested; age and BMI controlled (did not change results). Protective factors: none identified; authors suggest reducing appearance-focused Facebook content and following non-appearance pages as practical strategies	Objectification Theory (Fredrickson & Roberts, 1997); Social Comparison Theory (Festinger, 1954); Circle of Objectification (Strelan & Hargreaves, 2005); Temporal Comparison Theory (Albert, 1977)
S036	Song et al. (2025)	Appearance-based social comparison (state; post-exposure)		Appearance Comparison Scale (ACS; Tiggemann & McGill, 2004; 3 items: appearance thoughts, body fat/weight comparison, body shape thoughts; 7-point Likert 1=not at all to 7=very much; Cronbach's alpha=0.953 in this study)	No mediators tested (single DV study). ANOVA results: F(4,364)=4.144, p=0.003, partial eta-squared=0.044 (small-moderate effect). Post-hoc (LSD): all 4 model conditions (thin-ideal posed, thin-ideal functional, full-figured posed, full-figured functional) showed significantly higher ACS scores than scenery control (p-values 0.003	Moderators: none tested. BMI included as covariate in power analysis. Protective factors: Neutral scenery (non-human images) functioned as an effective protective condition — significantly lower appearance comparison than all model image types (scenery M=8.79, SD=4.72 vs. model groups M=10.72-11.99)	Social Comparison Theory (Festinger, 1954); Social Cognitive Theory (mentioned); Media Effects Theory; Cultivation Theory (cited as underutilized). Body positivity/body functionality framework (Alleva & Tylka, 2021)

ID	Author (Year)	Body image constructs	Self-esteem/wellbeing	Measurement instruments	Mediators	Moderators / protective	Framework
					to <0.001). No significant differences among the 4 model conditions (all p>0.1). H1 supported; H2 (thin vs. full-figured) NOT supported; H3 (posed vs. functional) NOT supported		
S037	Seekis et al. (2021)	Body dissatisfaction; drive for thinness		Fitspiration use: 5-item scale developed for study (1-5; alpha=0.89); Upward Physical Appearance Comparison Scale (UPACS; O'Brien et al., 2009; 10 items; 1-5; alpha=0.95); Body Dissatisfaction subscale EDI-3 (Garner, 2004; 10 items; 1-6; alpha=0.86); Drive for Thinness subscale EDI-3 (7 items; 1-6; alpha=0.92); Self-Compassion Scale (SCS; Neff, 2003; 26 items; 6 subscales: self-kindness, common humanity, mindfulness, reduced self-judgement, reduced isolation, reduced overidentification; 1-5); BMI as covariate	Mediation: upward appearance comparison (UPACS) mediated fitspiration to body dissatisfaction (B=0.084, 95% CI [0.043, 0.130]) and to drive for thinness (B=0.115, 95% CI [0.061, 0.174]). Moderated mediation: reduced uncompassionate facets (reduced self-judgement, isolation, overidentification) moderated the indirect path from fitspiration to body concerns via upward appearance comparison (H3/H4 partially supported). Compassionate facets (self-kindness, common humanity, mindfulness) + reduced isolation moderated the direct path from fitspiration to drive for thinness (H6 partially supported). No facets moderated the direct path to body dissatisfaction (H5 not supported)	Moderators: all 6 SCS facets tested as moderators. Significant buffering moderators found: reduced self-judgement, isolation, and overidentification (for indirect path via upward comparison); self-kindness, common humanity, mindfulness, reduced isolation (for direct path to drive for thinness). Protective factors: higher reduced self-judgement, isolation, and overidentification buffered fitspiration-upward comparison link; higher self-kindness, common humanity, mindfulness, and reduced isolation buffered fitspiration-drive for thinness link. Self-compassion (especially reduced uncompassionate responding) identified as key protective factor	Social Comparison Theory (Festinger, 1954); Objectification Theory (Fredrickson & Roberts, 1997; implicit); Self-Compassion Theory (Neff, 2003) - 6-facet model; Circle of Objectification (Strelan & Hargreaves, 2005; implicit)
S038	Lee (2022/2025)	Body satisfaction; drive for thinness		Instagram Addiction: Bergen Facebook Addiction Scale adapted for Instagram (Andreassen et al., 2012; 6 items; 1-5; alpha=0.89); Appearance Comparison: 3 items from Hendrickse et al. (2017) adapted from PACS (Thompson et al., 1991; 1-5; alpha=0.89); Objectification (body surveillance): OBCS Body Surveillance subscale	Serial mediation (SEM): Instagram addiction to body satisfaction via appearance comparison and objectification (beta=-0.10, 95% CI [-0.17, -0.03]; H5 supported). Instagram addiction drove thinness via appearance	Moderators: none tested. BMI included as covariate (negatively associated with body satisfaction, positively with drive for thinness). Protective factors: none identified; media literacy interventions and critical consumption of Instagram	Social Comparison Theory (Festinger, 1954); Objectification Theory (Fredrickson & Roberts, 1997); Integrated Social Comparison-Objectification model

ID	Author (Year)	Body image constructs	Self-esteem/wellbeing	Measurement instruments	Mediators	Moderators / protective	Framework
				(McKinley & Hyde, 1996; 8 items; 1-5; alpha=0.93); Body Satisfaction: MBSRQ-AE (Cash, 2000; 7 items; 1-5; alpha=0.92); Drive for Thinness: EDI subscale (Garner et al., 1983; 7 items; 1-5; alpha=0.94); BMI as covariate	comparison, objectification, and body satisfaction (beta=0.29, 95% CI [0.20, 0.37]; H6 supported). Direct paths: addiction to appearance comparison (beta=0.48, p<0.001); appearance comparison to objectification (beta=0.74, p<0.001); objectification to body satisfaction (beta=-0.35, p<0.001); objectification to drive for thinness (beta=0.39, p<0.001); body satisfaction to drive for thinness (beta=-0.13, p=0.01). Note: direct path addiction to objectification NOT significant (beta=0.06, ns)	suggested as practical strategies	
S039	Chang et al. (2019)	Body esteem		Body Esteem Scale (Mendelson, White, & Mendelson, 1996; original 20 items, reduced to 16 items via PCA; 1-5; Cronbach's alpha=0.93); Peer Appearance Comparisons: 3 items adapted from PACS (Thompson et al., 1991; 1-5; alpha=0.92); Direction of Comparisons: 2 items adapted from Fardouly & Vartanian (2015; -2=much worse to +2=much better; r=0.85); BMI as covariate	Full mediation by peer appearance comparisons: browsing to body esteem (indirect beta=-0.23, p<0.001; direct ns) and editing to body esteem (indirect beta=-0.13, p<0.01; direct ns). NO mediation for posting: selfie posting had DIRECT positive association with body esteem (beta=0.13, p<0.05) NOT mediated by peer appearance comparisons. Direction of comparisons (H3) NOT supported as moderator: no significant differences between worse-off group and equal/above group in path peer comparisons to body esteem	Moderators tested: direction of appearance comparisons (upward vs. lateral/downward) — NOT significant as moderator (H3 not supported). BMI as covariate (negatively associated with body esteem). Protective factors: selfie posting showed unexpected direct POSITIVE association with body esteem (especially in worse-off group, suggesting strategic image curation and peer support as possible mechanisms)	Objectification Theory (Fredrickson & Roberts, 1997; Calogero et al., 2011); Social Comparison Theory (Festinger, 1954; Wood, 1996); Self-presentation theory (Goffman, 1959; Hogan, 2010)
S040	Meier & Gray (2014)	Weight dissatisfaction; drive for thinness; thin ideal		Body-Esteem Scale for Adolescents and Adults - Weight Satisfaction subscale (BES; 8 items); Drive for	Not tested (correlational design); thin ideal internalization and	BMI controlled in all analyses; no moderators formally tested.	Tripartite Influence Model (media, peers, parents);

ID	Author (Year)	Body image constructs	Self-esteem/wellbeing	Measurement instruments	Mediators	Moderators / protective	Framework
		internalization; appearance comparison; self-objectification		Thinness subscale of Eating Disorder Inventory (EDI; 7 items); Sociocultural Internalization of Appearance Questionnaire-Adolescents (SIAQ-A; 5 items); Physical Appearance Comparison Scale (PACS; 4-item adapted version); Self-Objectification Questionnaire (SOQ; 10 items); Facebook Questionnaire (FBQ; 24 items) with Photo Subscale (8 items) to compute Appearance Exposure Score (AES)	appearance comparison discussed as theoretical mediators linking FB photo exposure to body dissatisfaction	Protective factors: not assessed	Objectification Theory (Fredrickson & Roberts, 1997); Social Comparison Theory (appearance comparison mechanism)
S041	Roberts et al. (2022)	Appearance esteem (proxy for body dissatisfaction); thin ideal internalization; muscular ideal internalization; body comparison (social appearance comparisons)		Sociocultural Attitudes Towards Appearance Questionnaire-4 (SATAQ-4): subscales for family pressure (4 items), peer pressure (4 items), traditional media pressure (4 items), social media pressure (4 items, adapted), thin ideal internalization (5 items), muscular ideal internalization (5 items); Body Comparison Orientation subscale of BEECOM (6 items, adapted for adolescents); Body Esteem Scale for Adolescents and Adults - Appearance subscale (BESAA; 10 items); BMI (self-reported height/weight)	Thin ideal internalization (mediates all 4 pressure sources -> appearance esteem, all p<.05); body comparison (mediates social media pressure -> appearance esteem, b=-0.16, p=.04); muscular ideal internalization (tested but not significant as mediator to appearance esteem in full model, p=.225)	Moderators: BMI and race/ethnicity (Latina reference group; White, Black, Asian, multiracial dummy codes) included as covariates on all paths. No protective factors formally tested	Adapted Tripartite Influence Model (Thompson et al., 1999) extended to include social media as 4th pressure source and muscular ideal internalization as additional mediator; Social Comparison Theory (Festinger, 1954)
S042	Scully et al. (2020)	Body dissatisfaction; frequency of social comparisons (with female targets: family, close friends, distal peers, celebrities); direction of social comparisons (upward comparisons); thin ideal internalization	self-esteem	Body Dissatisfaction subscale of Eating Disorder Inventory (EDI; Garner et al., 1983; 9 items, 0-45, alpha=.83); Thin-Ideal Internalization Scale (8 items, 0-40, alpha=.90); Rosenberg Self-Esteem Scale (10 items, 0-40, alpha=.78); Meier & Gray (2014) 8-item Photo Subscale (appearance activity, 0-32, alpha=.82); Fardouly & Vartanian (2015) items for comparison frequency (0-4) and direction/upward comparisons (1-7) with 4 female target groups	Serial multiple mediation: (1) frequency of social comparisons (significant mediator: appearance activity -> comparison frequency -> body dissatisfaction; b=.0697, 95% CI: .0129-.1416); (2) thin ideal internalization (significant serial mediator: appearance activity -> comparison frequency -> internalization -> body dissatisfaction; b=.0278, 95% CI: .0041-.0597). Upward comparisons and direct path of appearance activity on	Covariates: age and self-esteem (controlled in mediation model). No formal moderators tested. Protective factors: not assessed; future research suggested to examine body-positive content and media literacy as buffers	Sociocultural Model of Body Dissatisfaction (Thompson et al., 1999); Social Comparison Theory (Festinger, 1954); Objectification Theory (Fredrickson & Roberts, 1997)

ID	Author (Year)	Body image constructs	Self-esteem/wellbeing	Measurement instruments	Mediators	Moderators / protective	Framework
					body dissatisfaction not significant		
S043	Tiggemann et al. (2018)	Body dissatisfaction (weight + appearance dimensions); facial dissatisfaction; state appearance comparison; likes comparison (comparison on basis of number of likes received)		Visual Analogue Scales (VAS, 0-100mm) for body dissatisfaction (2 items: weight dissatisfaction + appearance dissatisfaction, averaged; alpha pre=.85, post=.90), facial dissatisfaction (1 item), and mood (5 items, not analyzed); State Appearance Comparison Scale (Tiggemann & McGill, 2004; 3 items, 1-7, alpha=.92) + 1 additional item for likes comparison; Instagram involvement measures (time, average likes, likes investment; 2-item investment scale alpha=.76); recall checks for likes and image thinness	State appearance comparison tested as predictor of post-exposure body/facial dissatisfaction (hierarchical regression): significant for body dissatisfaction overall (beta=.07, p=.033) but only in low-likes condition (beta=.14, p<.001); not significant in high-likes condition. Likes comparison did not predict body or facial dissatisfaction. No formal mediation model tested	Moderators tested (hierarchical regression): Instagram time, average likes received, likes investment — none moderated the effect of likes condition on body/facial dissatisfaction, appearance comparison, or likes comparison (all p>.10). Individual difference: likes investment (regardless of condition) positively correlated with appearance comparison (r=.26***), likes comparison (r=.39***), and facial dissatisfaction pre and post. Protective factors: not formally assessed	Social Comparison Theory (Festinger, 1954); Sociocultural Model of Body Dissatisfaction (Thompson et al., 1999); Social Reinforcement Theory (Thompson & Stice, 2001)
S044	Brown & Tiggemann (2016)	body dissatisfaction (weight dissatisfaction + appearance dissatisfaction + facial features dissatisfaction); state appearance comparison	Negative mood (5 dimensions: anxiety, depression, happiness, anger, confidence)	Visual Analogue Scales (VAS, 0-100mm) for negative mood (5 items, alpha=.85) and body dissatisfaction (3 items including facial, alpha=.94), pre and post exposure; State Appearance Comparison Scale (Tiggemann & McGill, 2004; 3 items, 1-7, alpha=.94); Celebrity Attitude Scale (CAS; McCutcheon et al., 2002; 17-item adapted version, 1-5, alpha=.91); Instagram usage questions (account, daily use, following); pilot-rated attractiveness/thinness/quality of stimuli	State appearance comparison: full mediation of image type on both negative mood (celebrity indirect: B=2.20, CI[0.93,3.95]; peer indirect: B=2.45, CI[1.12,4.37]) and body dissatisfaction (celebrity indirect: B=4.18, CI[2.29,7.17]; peer indirect: B=4.99, CI[2.79,7.82]). Direct effects of celebrity and peer images on mood and body dissatisfaction were non-significant after controlling for the mediator (PROCESS bootstrapping, 5000 samples)	Moderator: celebrity worship (CAS) moderated the effect of celebrity images (but not peer images) on body dissatisfaction (R2change=.01, Fchange(1,132)=4.98, p=.03): higher celebrity worship -> greater body dissatisfaction after viewing celebrity images. Celebrity worship also correlated with pre-exposure body dissatisfaction (r=.23, p=.01) and state appearance comparison (r=.23, p=.01). No protective factors assessed	Social Comparison Theory (Festinger, 1954); Sociocultural Model of Body Dissatisfaction (Thompson et al., 1999); Celebrity worship theory (Maltby et al., 2005)
S045	Cohen et al. (2017)	Thin-ideal internalisation; appearance comparison; appearance evaluation (satisfaction); body surveillance		SATAQ-3 Internalisation-General subscale (9 items, 1-5, alpha=.93); Physical Appearance Comparison Scale (PACS; 5 items, 1-5,	No mediation analysis conducted (correlational design). Appearance comparison and	BMI controlled in all correlations. No moderators formally tested. Protective factor	Sociocultural Theory of Body Image Disturbance (Thompson et al.,

ID	Author (Year)	Body image constructs	Self-esteem/wellbeing	Measurement instruments	Mediators	Moderators / protective	Framework
		(self-objectification); drive for thinness		alpha=.73); Appearance Evaluation subscale of MBSRQ (7 items, 1-5, alpha=.90); Body Surveillance subscale of OBCS (8 items, 1-7, alpha=.83); Drive for Thinness subscale of EDI-3 (7 items, 1-6, alpha=.89); Facebook Questionnaire (FBQ; Meier & Gray, 2014; 23 items, alpha total=.86, alpha PS=.71); Instagram account type frequency (3 single items: health & fitness, celebrity, travel; 1-5 scale)	appearance evaluation did not correlate significantly with appearance-focused SNS use; results discussed as consistent with prior work suggesting not all SNS activities relate to body image in the same way	implied: following appearance-neutral (travel) accounts on Instagram was not associated with any body image outcome (r range .04-.13, all ns), suggesting account type as a potential protective factor. Instagram users vs. non-users: users scored higher on body surveillance (t(243)=-2.18, p=.03, d=.30)	1999); Objectification Theory (Fredrickson & Roberts, 1997)
S046	McComb & Mills (2021)	State weight dissatisfaction; state appearance dissatisfaction; state confidence (all pre- and post exposure); cognitive emotion regulation strategies (rumination, catastrophizing, acceptance, positive refocusing)		Visual Analogue Scales (VAS, 0-100mm; Tiggemann & McGill, 2004 adapted) for appearance dissatisfaction, weight dissatisfaction, and confidence (pre and post, plus filler items); Physical Appearance Perfectionism Scale (PAPS; Yang & Stoeber, 2012) - worry about imperfection subscale only (7 items, 1-5, alpha=.87); Cognitive Emotion Regulation Questionnaire (CERQ; Garnefski et al., 2001) - 4 subscales adapted to comparison-to-Instagram context: rumination (alpha=.83), catastrophizing (alpha=.71), acceptance (alpha=.63), positive refocusing (alpha=.73); demographic questionnaire and BMI (measured objectively)	Cognitive coping strategies as mediators of PAPS-worry about imperfection -> body image (PROCESS model 4, experimental group only, n=72, 95% CI bootstrapping, alpha=.01): rumination and catastrophizing significantly mediated PAPS-WAI -> weight dissatisfaction (indirect: rumination b=.68 CI[.01,1.40]; catastrophizing b=.89 CI[.21,1.74]) and appearance dissatisfaction (rumination b=.63 CI[.09,1.04]; catastrophizing b=.70 CI[.19,1.56]) and confidence (rumination b=-.87 CI[-1.56,-.34]; catastrophizing b=-.83 CI[-1.45,-.26]). Positive refocusing mediated PAPS-WAI -> confidence (b=-.38). Acceptance: not a significant mediator for any outcome	Moderator: physical appearance perfectionism-worry about imperfection (PAPS-WAI) moderated the effect of experimental condition on weight dissatisfaction (B=1.73, p=.000), appearance dissatisfaction (B=1.20, p=.008), and confidence (B=-1.33, p=.004): medium/high perfectionism -> greater dissatisfaction and lower confidence after comparison. No effect in control condition. Protective factor: positive refocusing (adaptive coping) associated with lower appearance dissatisfaction and greater confidence	Social Comparison Theory (Festinger, 1954); Tripartite Influence Model (Rodgers et al., 2015); Cognitive Model of Body Dissatisfaction (Lewis-Smith et al., 2019); Clinical Perfectionism framework (Shafran et al., 2002; Fairburn et al., 2003)
S047	Fioravanti et al. (2024)	ED psychopathology (drive for thinness, bulimia, body dissatisfaction, interoceptive awareness); interpersonal	psychological investment in physical appearance	Instagram Use Questionnaire (IUQ; Kircaburun & Griffiths, 2019; Italian version; 9 items, 1-5, alpha=.87) measuring PIU; State	Serial mediation (SEM, MLR estimator, bootstrapped 95% CI, 10,000 resamples): PIU -	Age entered as covariate (significant for PIU and interpersonal difficulties). No formal moderators	Sociocultural Theory of Body Dissatisfaction (Thompson et al.,

ID	Author (Year)	Body image constructs	Self-esteem/wellbeing	Measurement instruments	Mediators	Moderators / protective	Framework
		difficulties (social insecurity, interpersonal distrust); body uneasiness (weight phobia, body image concerns, avoidance, compulsive self-monitoring, depersonalization); appearance comparison tendency		Appearance Comparison Scale (SACS; Tiggemann & McGill, 2004; Italian version; 3 items, 1-7, alpha=.94); Appearance Schema Inventory-Revised (ASI-R; Cash et al., 2004; 20 items, 1-5, alpha=.89); Body Uneasiness Test A (BUT-A; Cuzzolaro et al., 2006; 34 items, 0-5, GSI alpha=.97); Eating Disorder Inventory-2 (EDI-2; Garner, 1991; Italian version; 91 items, 11 subscales, overall alpha=.93); daily Instagram use (minutes)	> appearance comparison (SACS) -> body uneasiness (BUT-A) -> ED psychopathology (b=0.06, CI[0.01,0.11]); PIU -> psychological investment in appearance (ASI-R) -> body uneasiness -> ED psychopathology (b=0.12, CI[0.07,0.19]); PIU -> ASI-R -> ED psychopathology independently of BUT-A (b=0.03, CI[0.01,0.06]). Similar serial paths for interpersonal difficulties. SEM fit: CFI=0.987, TLI=0.968, RMSEA=0.054, SRMR=0.027	tested. ED patients vs. controls showed higher PIU, SACS, ASI-R, BUT-A and EDI-2 scores. Protective factors: not formally tested; social media literacy programs suggested as intervention target	1999); Social Comparison Theory (Festinger, 1954); Objectification Theory (Fredrickson & Roberts, 1997); Six-components model of behavioral addiction (Griffiths, 2005); PIU conceptualized as behavioral addiction
S048	Vendemia & Fox (2024)	State self-objectification (Twenty Statements Test, TST); animalistic dehumanization of peers (human uniqueness traits); mechanistic dehumanization of peers (human nature traits); appearance-related objectifying hashtags; sexually-objectifying hashtags; hashtag valence; hashtag categories (body parts, physical features, accessories/clothing/makeup, general appearance)		Twenty Statements Test (TST; Kuhn & McPartland, 1954; 20 "I am..." statements coded for physical appearance descriptors, kappa=.90; state self-objectification = sum of appearance items, range 0-20); Dehumanization via trait items from Haslam et al. (2005): human uniqueness (4 items, 1-6, alpha=.72; reverse-coded = animalistic dehumanization) and human nature (4 items, 1-6, alpha=.81; reverse-coded = mechanistic dehumanization); Hashtag coding scheme (Vendemia, DeAndrea & Brathwaite, 2021): appearance-related objectification (kappa=.95), sexual objectification (kappa=.74), 8-category appearance scheme (kappa=.93), valence (kappa=.91)	Post-hoc parallel mediation (PROCESS macro, Hayes 2022; image type dummy coded; 10,000 bootstrap resamples): generating body-parts hashtags mediated image type -> state self-objectification (non-sexualized: B=0.49, CI[0.01,1.24]; sexualized: B=2.78, CI[1.09,4.32]). No other hashtag categories (physical features, accessories, general appearance) served as significant mediators	Tagging vs. no-tagging condition: no significant difference on state self-objectification (main effect F(1,250)=.03, p=.86) or dehumanization (Wilks lambda p=.96). Image type was the key driver. Trait self-objectification not measured as a moderator (posttest-only design). Protective factors: not assessed; body-neutral/non-sexualized imagery implied as less harmful	Objectification Theory (Fredrickson & Roberts, 1997); distinction between sexual objectification and appearance-focused objectification (Morris et al., 2018); Dehumanization theory (Haslam, 2006: animalistic vs. mechanistic dehumanization); Social Comparison Theory (Festinger, 1954)
S049	Samson & Zaitsoff (2023)	Body satisfaction (pre-baseline and post-manipulation); appearance comparison (post-manipulation only)		Body Areas Satisfaction Scale (BASS; Brown et al., 1990; baseline alpha=.82; rated satisfaction with body areas on 5-point scale; administered pre and post); State Appearance Comparison Scale (SACS; Tiggemann & McGill, 2004;	Appearance comparison mediated image type -> changes in body satisfaction (PROCESS macro, Hayes 2018, Model 4; baseline body satisfaction as covariate;	No moderators formally tested. Groups did not differ on baseline body satisfaction, BMI, age, or Instagram use. 5 participants with past ED excluded from key	Social Comparison Theory (Festinger, 1954); Tripartite Influence Model (Thompson et al., 1999 cited); Narrative

ID	Author (Year)	Body image constructs	Self-esteem/wellbeing	Measurement instruments	Mediators	Moderators / protective	Framework
				alpha=.95; post only); demographics and self-reported daily Instagram use (minutes)	95% bootstrap CI): general fitspiration vs. control: b=-0.21, SE=0.05, CI[-0.32,-0.12]; body transformation vs. control: b=-0.18, SE=0.04, CI[-0.27,-0.11]. Direct effect of image type on body satisfaction non-significant after controlling for appearance comparison (p=.89; full mediation)	analyses (sensitivity analysis retained). Protective factors discussed: social media breaks (Lambert et al., 2022), self-compassion (Gobin et al., 2022), body-diversity accounts (Cohen et al., 2019); limiting fitspiration/transformation exposure recommended	Transportation Theory (Green & Dill, 2013; Perloff, 2014) as theoretical framework for transformation imagery
S050	Rousseau (2024)	Body surveillance; body appreciation		Body Surveillance subscale - Objectified Body Consciousness Youth Scale (Lindberg et al., 2006), 4 items, 5-point scale (alpha=.82); Body Appreciation Scale-2 Dutch version (BAS-2; Tylka & Wood-Barcalow, 2015a; Alleva et al., 2016), 10 items, 5-point scale (alpha=.92)	Upward appearance comparisons on social media (adapted UPACS, O'Brien et al., 2009; 10 items, alpha=.91); Broad conceptualization of beauty (Broad Conceptualization of Beauty Scale, Tylka & Iannantuono, 2016; 9 items, 7-point scale, alpha=.77)	Age, BMI, frequency of Instagram use (controls). No moderators tested.	Social Comparison Theory (Festinger, 1954); Objectification Theory (Fredrickson & Roberts, 1997); Acceptance Model of Body Appreciation and Intuitive Eating (Avalos & Tylka, 2006)
S051	Markey & Daniels (2022)	Body esteem; body appreciation; self-objectification (body surveillance, body shame); broad conceptualization of beauty		Body Appreciation Scale-2 (BAS-2; Tylka & Wood-Barcalow, 2015), 10 items, 5-point scale (T1 alpha=.93, T2 alpha=.95); Objectified Body Consciousness Scale for Youth (OBC-Y; Lindberg et al., 2006): Surveillance subscale 4 items + Shame subscale 5 items, 7-point scale; Body Esteem Scale modified (BES; Mendelson et al., 2001), 21 items, 5-point scale (T1 alpha=.92); Broad Conceptualization of Beauty Scale (7 items adapted; Tylka & Iannantuono, 2016), 7-point scale (T1 alpha=.66)	None formally tested (no mediation analysis)	Type of engagement (appearance-focused vs. communication-focused) as key moderating variable; age and platform use as covariates; no significant ethnic/racial group differences found	Objectification Theory (Fredrickson & Roberts, 1997) - implicit; no explicit theoretical framework stated
S052	Prichard et al. (2023)	state body dissatisfaction; state appearance comparison; state self-objectification	State negative mood	Visual Analogue Scales (VAS; Heinberg & Thompson, 1995): negative mood (5 items, alpha pre=.79, post=.82) and body dissatisfaction (4 items, alpha pre=.86, post=.88); State Appearance Comparison Scale (Tiggemann & McGill, 2004), 3 items, 7-point scale (alpha=.92); modified Twenty Statements Test	State appearance comparison (significant mediator); state self-objectification (tested but not a significant mediator). Parallel mediation via PROCESS Model 4 (10,000 bootstrap samples)	BMI, age, baseline mood/body dissatisfaction as covariates. No moderators tested. Degree of sexualization as experimental manipulation.	Sociocultural Theory (Thompson et al., 1999); Objectification Theory (Fredrickson & Roberts, 1997)

ID	Author (Year)	Body image constructs	Self-esteem/wellbeing	Measurement instruments	Mediators	Moderators / protective	Framework
				(Fredrickson et al., 1998), 10 items coded for appearance-based responses			
S053	Patel et al. (2025)	State appearance comparison; urge to use appearance-related safety behaviors; overvaluation of appearance (self-report + task-based)	Appearance concerns (social appearance anxiety); state sadness	State Appearance Comparison Scale (SACS; Tiggemann & McGill, 2004), 3 items, 7-point scale (alpha=.83); Social Appearance Anxiety Scale (SAAS; Hart et al., 2008), 16 items, 5-point scale (alpha=.96); Appearance-Related Safety Behavior Scale (ARSBs; Patel et al., 2022), 13 items adapted as urge measure, 5-point scale (alpha=.81); State sadness single item, 4-point scale; Beliefs About Appearance Scale (BAAS; Spangler & Stice, 2001), 20 items (alpha=.96); Appearance Preference Task (APT; Patel et al., 2023), forced-choice computerized task (alpha=.96)	None formally tested (no mediation analysis)	Order of video presentation (counterbalanced; no significant order effects found). Baseline appearance concerns/mood not assessed as moderator (limitation noted).	Sociocultural Theory / Tripartite Influence Model (implicit); Social Comparison Theory (Festinger, 1954)
S054	Tiggemann & Barbato (2018)	State body dissatisfaction; state self-objectification		Visual Analogue Scales (VAS; Heinberg & Thompson, 1995): body dissatisfaction pre/post (2 items: weight dissatisfaction + appearance dissatisfaction, 0-100 scale; pre alpha=.87, post alpha=.90); modified Twenty Statements Test (Fredrickson et al., 1998), 10 items coded for appearance-based responses (state self-objectification); Self-Objectification Questionnaire (Noll & Fredrickson, 1998), trait measure	None formally tested (no mediation analysis)	Trait self-objectification as potential moderator (tested via hierarchical regression; significant independent predictor of body dissatisfaction but did not moderate effect of comment type). BMI, age, Instagram use as covariates.	Objectification Theory (Fredrickson & Roberts, 1997)
S055	Pryde & Prichard (2022)	State body dissatisfaction; state appearance comparison	state negative mood	Visual Analogue Scales (VAS; Heinberg & Thompson, 1995): body dissatisfaction (4 items, 0-100; baseline alpha=.85, post alpha=.92) and negative mood (5 items; baseline alpha=.72, post alpha=.72); State Appearance Comparison Scale (SACS; Tiggemann & McGill, 2004), 3 items, 7-point scale (alpha=.92); Fit Ideal Internalisation Test (FIIT; Uhlmann et al., 2020), 20 items, 5-point scale (alpha=.94)	State appearance comparison (significant full mediator of TikTok condition on body dissatisfaction and negative mood; PROCESS Model 4, 10,000 bootstrap samples)	Trait fit ideal internalisation as moderator (tested via PROCESS Model 1; not significant). Baseline body dissatisfaction/mood as covariates.	Sociocultural Theory (Tiggemann, 2011b); Social Comparison Theory (Festinger, 1954)

ID	Author (Year)	Body image constructs	Self-esteem/wellbeing	Measurement instruments	Mediators	Moderators / protective	Framework
S056	Becker et al. (2025)	Body dissatisfaction (negative body image); body appreciation (positive body image)		Modified Instagram Photo Activity Index (Meier & Gray, 2014), 9 items, 7-point scale (alpha=.85 each content type); Online Physical Appearance Comparison Scale (Online PACS), 4 items, 5-point scale (alpha=.79); Thin-Internalisation Subscale SATAQ-4 (Schaefer et al., 2015), 5 items, 5-point scale (alpha=.80); Body Acceptance by Others Scale-2 (BAOS-2; Swami et al., 2021), 13 items, 5-point scale (alpha=.94); Body Surveillance Subscale OBCS (McKinley & Hyde, 1996) as internal body orientation, 8 items, 7-point scale (alpha=.82); MBSRQ Body Areas Satisfaction Subscale (Cash, 1990), body dissatisfaction (alpha=.76); BAS-2 (Tylka & Wood-Barcalow, 2015b), 10 items, 5-point scale (alpha=.93)	Appearance comparison (not significant); thin-ideal internalisation (significant for body dissatisfaction only); body acceptance by others (significant for both); internal body orientation (significant for both). SEM via Mplus, bootstrapping 1000 resamples	No moderators tested. Age, education, country as demographic descriptors.	Tripartite Influence Model (Thompson et al., 1999); Acceptance Model of Intuitive Eating (Avalos & Tylka, 2006)
S057	Engeln et al. (2020)	State body satisfaction; appearance comparisons; appearance-focused thoughts	positive affect; negative affect	Body Image States Scale (BISS; Cash et al., 2002), 6 items, 9-point scale (pre alpha=.80, post alpha=.82); PANAS brief version (Watson et al., 1988), 10 items PA + 10 items NA, 5-point scale (PA alphas .86/.91; NA alphas .87/.87); State appearance comparison measure (adapted from Tiggemann & McGill, 2004), 2 items + 1 appearance-focus item, 7-point scale (alpha=.83)	None formally tested (no mediation analysis)	Platform type (Instagram vs. Facebook vs. control) as key experimental variable. No moderators formally tested; trait appearance comparison not measured.	Social Comparison Theory (Festinger, 1954)
S058	Anixiadis et al. (2019)	State body dissatisfaction	state negative mood	Visual Analogue Scales (VAS; Heinberg & Thompson, 1995): body dissatisfaction (3 items, 0-100; alpha=.81) and negative mood (5 items; alpha=.80); Physical Appearance Comparison Scale-Revised (PACS-R; Schaefer & Thompson, 2014), 11 items, 1-5 scale (alpha=.87) - trait; SATAQ-4 Thin-Internalisation subscale (Schaefer et al., 2015), 5 items, 1-5 scale (alpha=.78) - trait; Thought listing (qualitative coding: upward body comparisons, media literacy, positive body judgements, other-focus thoughts)	State upward body comparison thoughts (qualitative, thought listing - unique predictor of mood change); positive body judgements (associated with mood change but not unique); other-focus thoughts (protective but not unique after accounting for upward comparisons)	Trait appearance comparison tendencies and trait thin-ideal internalisation as moderators (both marginal/non-significant). BMI, age, education as demographics.	Sociocultural Theory (Thompson et al., 1999); Media Literacy Model (McLean et al., 2016a)

Note. Studies are identified by Study ID (S001–S058) and first author with year of publication. Full citations are listed in the reference list of the main manuscript.
