# Supplementary material for: Bodies on Display: A Scoping Review of Appearance-Focused Social Media, Body Image, Self-Esteem, and Psychological Wellbeing in Adolescent Girls and Young Women
Source: Eur J Investig Health Psychol Educ. 2026 Jul 23;16(8):108. doi: 10.3390/ejihpe16080108 (PMC13512515; doi:10.3390/ejihpe16080108)
Supplement: Supplementary file 1 [file ejihpe-16-00108-s001.zip › Table_S4_PRISMA_ScR_Checklist.pdf]

Table S4. PRISMA-ScR Checklist

Bodies on Display: A Scoping Review of Appearance-Focused Social Media, Body Image, Self-Esteem, and Psychological Wellbeing in Adolescent Girls and Young Women

The present scoping review was reported in accordance with the Preferred Reporting Items for Systematic Reviews and Meta-Analyses extension for Scoping Reviews (PRISMA-ScR) checklist (Tricco et al., 2018). The table below details how each of the 22 checklist items has been addressed.

Section	Item	PRISMA-ScR Checklist Item	Reported in Section / Table
**TITLE**	**1**	Identify the report as a scoping review.	Title page; the title identifies the report as a scoping review.
**ABSTRACT**	**2**	Provide a structured summary that includes (as applicable): background, objectives, eligibility criteria, sources of evidence, charting methods, results, and conclusions that relate to the review questions and objectives.	Abstract.
**INTRODUCTION**	**3**	Describe the rationale for the review in the context of what is already known. Explain why the review questions/objectives lend themselves to a scoping review approach.	Sections 1.1–1.4.
	**4**	Provide an explicit statement of the questions and objectives being addressed with reference to their key elements (e.g., population or participants, concepts, and context) or other relevant key elements used to conceptualize the review questions and/or objectives.	Section 1.5.
**METHODS**	**5**	Indicate whether a review protocol exists; state if and where it can be accessed (e.g., a Web address); and if available, provide registration information, including the registration number.	Section 3.1; protocol registered on the Open Science Framework (OSF; registration DOI: https://doi.org/10.17605/OSF.IO/BJZGX).
	**6**	Specify characteristics of the sources of evidence used as eligibility criteria (e.g., years considered, language, and publication status), and provide a rationale.	Section 3.2.
	**7**	Describe all information sources in the search (e.g., databases with dates of coverage and contact with	Section 3.3; PubMed, Scopus, Web of Science. Search conducted April–May 2026.

Section	Item	PRISMA-ScR Checklist Item	Reported in Section / Table
		authors to identify additional sources), as well as the date the most recent search was executed.	
	**8**	Present the full electronic search strategy for at least 1 database, including any limits used, such that it could be repeated.	Section 3.3; full search strings in Table S1.
	**9**	State the process for selecting sources of evidence (i.e., screening and eligibility) included in the scoping review.	Section 3.4.
	**10**	Describe the methods of charting data from the included sources of evidence (e.g., calibrated forms or forms that have been tested by the team before their use, and whether data charting was done independently or in duplicate) and any processes for obtaining and confirming data from investigators.	Section 3.5.
	**11**	List and define all variables for which data were sought and any assumptions and simplifications made.	Section 3.5; charted variables listed (i)–(ix).
	**12***	If done, provide a rationale for conducting a critical appraisal of included sources of evidence; describe the methods used and how this information was used in any data synthesis (if appropriate).	Section 3.5; no formal critical appraisal undertaken, in line with scoping review methodology (Peters et al., 2020; Tricco et al., 2018). Not applicable.
	**13**	Describe the methods of handling and summarizing the data that were charted.	Section 3.6.
**RESULTS**	**14**	Give numbers of sources of evidence screened, assessed for eligibility, and included in the review, with reasons for exclusions at each stage, ideally using a flow diagram.	Section 4.1; Figure 1.
	**15**	For each source of evidence, present characteristics for which data were charted and provide the citations.	Section 4.2; Table 1; Table S2.
	**16***	If done, present data on critical appraisal of included sources of evidence (see item 12).	Not applicable (no critical appraisal conducted).

Section	Item	PRISMA-ScR Checklist Item	Reported in Section / Table
	**17**	For each included source of evidence, present the relevant data that were charted that relate to the review questions and objectives.	Sections 4.3–4.5; Tables S2 and S3.
	**18**	Summarize and/or present the charting results as they relate to the review questions and objectives.	Sections 4.2–4.5.
**DISCUSSION**	**19**	Summarize the main results (including an overview of concepts, themes, and types of evidence available), link to the review questions and objectives, and consider the relevance to key groups.	Sections 5.1 and 5.2.
	**20**	Discuss the limitations of the scoping review process.	Sections 5.3 and 5.4.
	**21**	Provide a general interpretation of the results with respect to the review questions and objectives, as well as potential implications and/or next steps.	Section 6.
**FUNDING**	**22**	Describe sources of funding for the included sources of evidence, as well as sources of funding for the scoping review. Describe the role of the funders of the scoping review.	To be completed by author.

Note. * Items 12 and 16 (critical appraisal of individual sources of evidence) are optional within the PRISMA-ScR framework and were not addressed, in line with the mapping objective of scoping review methodology (Peters et al., 2020; Tricco et al., 2018). Page numbers may be added once the manuscript is paginated for submission.

Source: Tricco AC, Lillie E, Zarin W, et al. PRISMA Extension for Scoping Reviews (PRISMA-ScR): Checklist and Explanation. Ann Intern Med. 2018;169(7):467-473. doi:10.7326/M18-0850
